# Janus Nanoparticles by Pickering Emulsions: A Versatile Approach for the Selective Functionalization of SiO_2_

**DOI:** 10.1021/acsami.5c20303

**Published:** 2026-01-28

**Authors:** Elisa Manzini, Silvia Mostoni, Massimiliano D’Arienzo, Luciano Tadiello, Roberto Scotti, Barbara Di Credico

**Affiliations:** † Department of Materials Science, INSTM, 9305 University of Milano-Bicocca, Via Roberto Cozzi, 55, Milan 20125, Italy; ‡ 433522 Pirelli Tyre S.P.A, Viale Piero E Alberto Pirelli, 25, Milan 20126, Italy

**Keywords:** Janus nanoparticle, pickering, emulsion, silica, selective functionalization, silane, interface

## Abstract

Janus nanoparticles (JNPs) are fascinating colloidal-sized particles characterized by different properties on opposite sides. Among the synthetic approaches, the preparation of JNPs by a masking approach with paraffin wax in water Pickering emulsions appears to be a flexible method for the selective modification of SiO_2_ NPs. However, the preparation of JNPs by Pickering emulsions at the nanometric level has yet to be investigated. With the aim of extending the applicability of the method to the modification of nanofillers, commonly employed in polymer composites, the preparation of Pickering emulsions with SiO_2_ and paraffin wax has been rationalized. In detail, starting from micrometric silica, a procedure valid for particles of different sizes down to 30 nm was established for the first time. The NPs were functionalized on one hemisphere with (3-aminopropyl)­triethoxysilane (APTES), and the resulting APTES-JNPs were further modified with commercially available polymers. The polymer-JNPs show anisotropic characteristics that could impart interesting properties for different applications and could be further modified to create specific building blocks.

## Graphical abstract

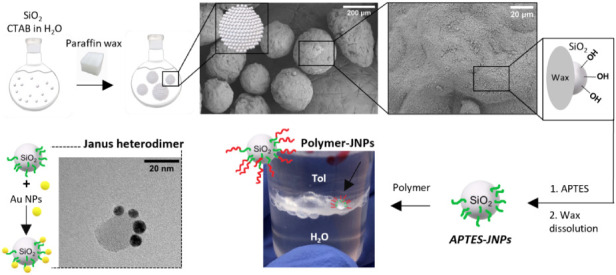

## 1 Introduction

During the past decade, the possibility of designing nanoparticles (NPs) of hybrid nature by combining organic and inorganic components in a synergistic way has raised a lot of interest in the scientific community.^1,2^ However, as the material requirements become more and more restrictive and specific, it becomes evident that there is a necessity to exert higher control over their synthesis to produce engineered NPs with desired functionalities.

Among hybrid NPs, a particularly high level of selectivity in the NPs’ modification is required for the synthesis of Janus NPs (JNPs),^3^ named after the Roman god Janus, who has two different faces.^4^ In fact, JNPs are colloid-sized particles with two regions of different surface chemical compositions, providing unique asymmetric characteristics and properties to the single NPs. Many efforts have been made for the preparation of JNPs having silica as the body, due to its unique properties and applications in several fields ranging from photocatalysis, optics, and electronics to chemical reactivity in confined spaces and biological applications.^5–10^

Janus particles of different nature and dimensions, ranging between 40 nm and 1.5 μm, were developed and exploited as (i) efficient compatibilizers of immiscible phases (by acting analogously to surfactants or copolymers),^11–13^ (ii) fiber coatings,^14,15^ (iii) self-assembled materials,^16,17^ (iv) catalysts,^18–21^ and (v) materials for drug delivery.^22^

However, despite their high potential, the major challenge ahead is still the preparation of Janus particles in large quantities and with nanometric dimensions, considering that the more available and effective the approaches to modify matter at the nanometric level are, the higher the possibility will be to have control over the final material and its potential application.

Among the preparation methods of silica JNPs^23,24^ the most employed is the masking approach,^25–29^ by which JNPs are obtained through a *masking* step in which particles are trapped at the interface between two phases, allowing surface modification only on one NP side. The method is highly flexible, given the possibility to modify materials of different natures with proper functionalizing agents. The main difficulties lie in the selection of the appropriate interface (hard or soft substrates, emulsions) to achieve good selectivity and in scaling up the preparation method. In this context, Granick et al.^25,30^ developed an interesting approach for the selective functionalization of NPs by controlling their localization at an oil/water (o/w) interface with Pickering emulsions, a viable approach for colloidosome preparation.^31,32^

The procedure for the preparation of JNPs consists of making an o/w Pickering emulsion with paraffin wax as the oil phase. Once the emulsion is formed, the temperature is lowered to solidify the wax, thus blocking the particles’ rotational movements at the interface between the wax and water. As a result, the adsorbed particles have only one exposed hemisphere, which can be modified by finding the best functionalization conditions.

In their first attempt, the Granick group^25^ employed fused SiO_2_ particles with diameters of 800 nm and 1.5 μm, which were functionalized in solvents with (3-aminopropyl)­triethoxysilane (APTES) on one side and with n-octadecanetrichlorosilane on the second hemisphere. However, as mentioned by Granick et al.,^33^ the employment of solvents for the surface modification often causes the particles’ detachment from the wax colloidosomes. Therefore, the same group investigated the possibility of modifying the SiO_2_ exposed surface with dichlorodimethylsilane and aminopropyldimethylethoxysilane by vapor deposition at room temperature (rt), obtaining a fast and successful chemical modification.^33^

Despite the high potential of this JNPs preparation approach, only a few examples in the literature report the preparation of Pickering emulsions with particles having a core size below 100 nm, as it is more difficult to immobilize smaller particles at the interface and to obtain a good emulsion with high stability from a thermodynamic point of view. Although sub-100 nm Janus particles have been reported, prior studies do not describe wax-based Pickering emulsions stabilized by silica particles below this size range, nor do they address the controlled formation of JNPs via selective adhesion onto a wax template.^26,30,34^ To the best of our knowledge, wax Pickering emulsions using significantly smaller silica NPs for Janus fabrication have not been reported.

In addition, several different formulations in terms of solvent, surfactants, and other additives have been reported for the generation of wax Pickering emulsions, making the data rationalization complex. Some studies report the use of water as a solvent, while others employ alcohol solutions (methanol, ethanol). The addition of surfactants (i.e., cetyltrimethylammonium bromide (CTAB), didodecyldimethylammonium bromide) to partially hydrophobize SiO_2_ NPs is often described,^26^ especially in the case of hydrophilic silica particles.^35^ In addition, in most cases, clear indications about the impact of SiO_2_ NPs, wax, and surfactant amounts and ratios on paraffin wax-water Pickering emulsions are missing.

To sum up, previous Pickering emulsion–based protocols for the preparation of Janus particles^25,33,34^ were generally developed for a single particle size and do not provide clear guidelines for adapting the methodology to variations in particle dimensions and silica surface properties, such as surface hydroxyl density.

To face these issues, a better knowledge of silica-wax Pickering emulsion preparation and formation is mandatory.

In this context, the present work aims to establish a procedure for the preparation of paraffin wax-in-water Pickering emulsions with SiO_2_ particles of varying sizes and, once optimized, to apply this method for the selective functionalization of NPs. In detail, after an extensive investigation of the surface features of the SiO_2_ particles synthesized by the Stöber method,^36^ the preparation of Pickering emulsions of silica and paraffin wax was optimized by changing several parameters to obtain a stable wax-in-water emulsion. In particular, starting from the analysis of the Pickering emulsions with micrometric silica, the same approach was extended to particles of nanometric dimensions (<100 nm). Specifically, the micrometric silica particles were first used as a model system to fine-tune the key synthesis parameters (particularly surfactant concentration, mixing conditions, and interfacial stabilization) because their size facilitates more straightforward observation and characterization of the Pickering emulsion behavior. By optimizing the experimental parameters, complete silica coverage of paraffin wax colloidosomes was obtained regardless of the quantities scaled up. Then, their adsorption stability was evaluated in order to determine the appropriate functionalization conditions. For this step, a common functionalizing agent, APTES, was used, and good selectivity was attained, resulting in the desired APTES-functionalized JNPs (APTES-JNPs).

The effective functionalization of only one hemisphere of silica NPs was confirmed, thanks to the preparation of a Janus heterodimer consisting of a core of silica NP decorated with gold NPs only on the functionalized hemisphere, as clearly shown by morphological analysis. This Janus heterodimer is of certain interest, combining broadly different properties provided by two dissimilar inorganic materials. In addition, starting from dipolar APTES-JNPs, the selective grafting of commercially available polystyrene (PS) and polybutadiene (PB) oligomers having succinic anhydride functionalities was investigated.^6^

Therefore, in this work, building on Granick’s established approach, we introduced a systematic optimization strategy that facilitates the extension of the method according to experimental requirements. Specifically, the zeta potential is used as a quantitative parameter to guide the selection of the CTAB concentration required to stabilize the emulsion interface, enabling the reproducible preparation of JNPs in the nanometric size range and access to particle sizes smaller than those typically reported.

In addition, the stability of the colloidosomes was evaluated in different EtOH/H_2_O ratios to ensure compatibility with the subsequent functionalization step, further improving the robustness and versatility of the protocol.

## 2 Materials and Methods

### 2.1 Materials

Tetraethoxysilane (TEOS, ≥99%), 25% aqueous ammonia solution, HCl 37%, paraffin wax (melting point 53–58 °C), (3-aminopropyl)­triethoxysilane (APTES, ≥98%), gold NPs (5 nm, OD 1, stabilized suspension in citrate buffer, 4.92–6.01 × 10^13^ particles/mL), poly­(styrene-*co*-maleic anhydride) cumene terminated (*M*_n_ ≈ 1900 g mol^–1^, PS) were purchased from Sigma-Aldrich. Toluene (99%) and cetyltrimethylammonium bromide (CTAB, ≥98%) were purchased from Alfa Aesar. Ethanol absolute (EtOH ≥99.8%) was purchased from Honeywell. Liquid maleated polybutadiene (Polyvest MA75, *M*_n_ ≈ 3000 g mol^–1^, PB) was purchased from Evonik. Milli-Q water with a resistivity ρ > 18.2 MΩ·cm was used.

### 2.2 Synthesis of Silica Particles

In order to obtain batches of monodispersed silica particles of different dimensions, Stöber conditions were employed by tuning the temperature as well as the reactant concentrations .

For the synthesis of the micrometric silica particles, 85 mL of EtOH and 40 mL of distilled water were added to a 250 mL flask, and the mixture was stirred at 1000 evolutions per minute (rpm). Then, 30 mL of TEOS were slowly poured in, and after 30 min, 8 mL of NH_4_OH were added to the flask. The concentrations of the reactants were as follows : TEOS 0.82 M, H_2_O 15.5 M, NH_4_OH 0.65 M. The reaction mixture was stirred (1000 rpm) at rt for 45 min, and then 10 mL of a 2 M HCl solution were added to induce the silica NPs’ flocculation and precipitation. For the retrieval of silica particles, the dispersion was centrifuged and then washed four times with ethanol and water. The sample was dried at 80 °C overnight and named SiO_2_-μm.

Silica NPs were prepared according to the procedure reported elsewhere,^6^ as described in the Supporting Information (SI, Paragraph S1). The sample was called SiO_2_-nm.

### 2.3 Preparation of the SiO_2_–Paraffin Wax Pickering Emulsion

SiO_2_ particles were dispersed in 30 mL of an aqueous CTAB solution in a 50 mL round flask (Scheme 1). The dispersion was sonicated for 20 min and then heated to 80 °C. Paraffin wax was then added gradually into the dispersion, and after all the wax had melted, the mixture was subjected to vigorous magnetic stirring (1100 rpm). After 2 h, the emulsion was cooled to rt. To retrieve the colloidosomes, the emulsion was filtered, washed with water, and then dried at rt. The preparation procedure was the same for both silica particles. The optimized amounts of silica, paraffin wax, and CTAB used for the successful formation of Pickering emulsions with both silica particles are reported in Table 1 (the values will be discussed below in Paragraph 3.1). This method was confirmed to be scalable up to 0.4 g of particles by maintaining the same SiO_2_/wax and CTAB/SiO_2_ ratios, with no significant change in the resulting Pickering emulsion quality.

**Table 1:** Experimental Data for the Preparation of SiO_2_-μm and SiO_2_-nm Pickering Emulsions

Sample	SiO_2_ (g)	Paraffin Wax (g)	CTAB (g/L)
SiO_2_-μm	0.1	3.00	0.01095
SiO_2_-nm	0.1	0.63	0.2192

**Scheme 1:**
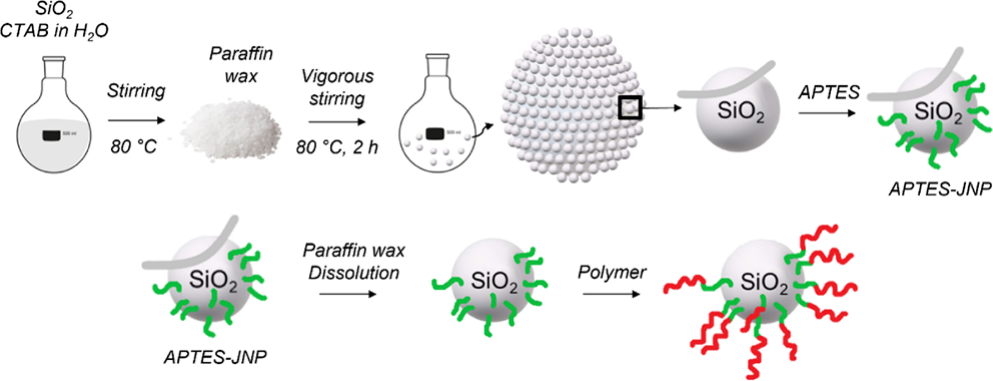
Schematic Representation of the APTES-JNP Preparation.^a^

a At first, SiO_2_-paraffin wax colloidosomes were formed by the preparation of a Pickering emulsion. The particles immobilized at the water/paraffin wax interface were functionalized with APTES only on one hemisphere. Starting from APTES-JNPs, polymer-JNPs were prepared in order to confirm their selective functionalization.

### 2.4 Functionalization of Simplified NPs with APTES

In order to introduce amino groups as anchoring points for the subsequent grafting of polymer chains, silica NPs (SiO_2_-nm) were functionalized with APTES. Starting from the Pickering emulsion (0.2 g of SiO_2_ and 1.26 g of paraffin wax , Table 1), the colloidosomes were dispersed in a 50/50 EtOH/water solution (40 mL) by magnetic stirring at 500 rpm at rt for 20 min. Afterward, APTES was added, and the reaction was carried out for 24 h at rt. After 24 h, the pH of the mixture was adjusted to 2–3 to induce the colloidosomes’ precipitation, and the dispersion was centrifuged at 9000 rpm for 30 min and then at 3000 rpm for 10 min. The solid was washed two times with 30 mL of EtOH to remove both unreacted species and CTAB, and centrifuged at 9000 rpm for 20 min. To dissolve the paraffin wax, the colloidosomes were redispersed in 30 mL of toluene at rt, sonicated, and then centrifuged for 10 min at 9000 rpm. The washing step with toluene was repeated a second time. The dipolar Janus NPs, APTES-JNPs, were dried at 80 °C overnight.

### 2.5 Grafting of Polymer Chains on Silica JNPs

To prepare Polymer-JNPs, the APTES-JNPs (Scheme 1) were dispersed by sonication in 20 mL of toluene, and the dispersion was heated to 120 °C and stirred at 350 rpm. After reaching the solvent’s boiling temperature, the polymer (PS or PB) was added to the flask in a Polymer:APTES ratio of 1:2. In the case of PB, the polymer was previously dissolved in 5 mL of toluene for a total volume of 20 mL.

The reaction was performed for 24 h, and then the dispersion was cooled down at rt and centrifuged for 15 min at 9000 rpm. The retrieved NPs were washed two times with 30 mL of toluene, sonicated, and then centrifuged for 15 min at 9000 rpm. The samples, namely PS-JNPs and PB-JNPs, were dried at 80 °C overnight.

### 2.6 Morphological, Structural, and Spectroscopic Characterization

Silica particles were morphologically characterized by transmission electron microscopy (TEM, JEOL JEM-2100+, acceleration voltage of 200 kV). The particles were ground into a fine powder, dispersed in EtOH, and two drops of the suspension were deposited on a 3 nm carbon-coated Cu TEM mesh grid.

A volume of 900 μL of the silica particle dispersion, prepared by dispersing 20 mg of powder in 2 mL of H_2_O by sonication, was analyzed by dynamic light scattering (DLS) using a Malvern Zetasizer Nano S instrument. The results of the DLS analysis were provided as number- and intensity-based distributions.

Chemical surface modification of silica samples was studied by attenuated total reflectance (ATR)–Fourier transform infrared spectroscopy (FTIR), performed on a Thermo Fisher Nicolet iS20 FTIR spectrometer with 4 cm^–1^ resolution, 525–4000 cm^–1^ region, 32 scans.

CHNS elemantal analyses were conducted with an Analyzer Elementar VarioMICRO.

Thermogravimetric analysis (TGA, Mettler Toledo TGA/DSC1 STARe System) was performed with a constant air flow (50 mL min^–1^) by (i) a heating ramp from 30 to 150 °C at a rate of 10 °C min^–1^, (ii) an isotherm at 150 °C for 10 min (to remove any residual solvent or adsorbed water), and then (iii) a second heating ramp until 1000 °C at a heating rate of 10 °C min^–1^.

MicroActive TriStar II Plus apparatus was utilized to record the N_2_ physisorption isotherm on silica samples (at 77 K in a liquid N_2_ bath). Brunauer–Emmett–Teller (BET, ASAP 2020 Plus) surface area analysis^37^ was applied to measure the specific surface area (SSA) after evacuation of the samples at 100 °C for 12 h. The total pore volume was obtained from the maximum nitrogen adsorbed volume at *p*/*p*_0_ = 1 using the instrument software.

To determine the appropriate conditions for the formation of a Pickering emulsion, ζ-potential measurements (Malvern Zetasizer Nano Series ZS90) were performed on bare and CTAB-adsorbed silica particles under the same pH conditions applied for the preparation of the Pickering emulsion. The recorded values were interpolated using the Akima Spline function in order to obtain a better estimation of the CTAB concentrations corresponding to low absolute surface charge values.

To confirm the colloidosomes’ formation as well as the effective localization of SiO_2_ on the wax spheres’ surface and analyze the changes in the colloidosomes’ dimensions over time, the emulsions were investigated using optical microscopy (OM, Olympus BX51 TRF) and scanning electron microscopy (SEM, Zeiss Gemini SEM 500 microscope) analyses. To measure the colloidosomes’ and particles’ diameters , the ImageJ processing program (Image Processing and Analysis in Java) was employed. The polydispersity index (PDI) value was calculated as $\sqrt{\left(\sigma /d\right)}$ where *σ* is the standard deviation and *d* the average diameter. To perform SEM analysis, the dried colloidosomes were redispersed in different H_2_O/EtOH ratios (100, 50/50, 70/30, and 0) to investigate both their structure and miscibility and to confirm their integrity for the subsequent silane-functionalization. The sample was prepared by depositing a few drops of the prepared solutions until the formation of a visible layer of colloidosomes was observed. All the samples, prior to SEM analysis, were gold-sputtered.

To verify the selective grafting of PS- and PB-silica NPs, the latter were dispersed by sonication in a mixture of water and toluene (50/50), and their localization was visually analyzed.

## 3 Results and Discussion

### 3.1 Surface Properties Characterization of Silica Particles

In order to set up the Pickering emulsions procedure, two different sizes of silica particles (SiO_2_-μm and SiO_2_-nm) were synthesized by Stöber method and their surface features were deeply characterized by morphological, thermogravimetric, spectroscopic, and ζ-potential analyses (as reported in Figure 1 and Tables S1, S2, S3 of Paragraph S1).

**Figure 1:**
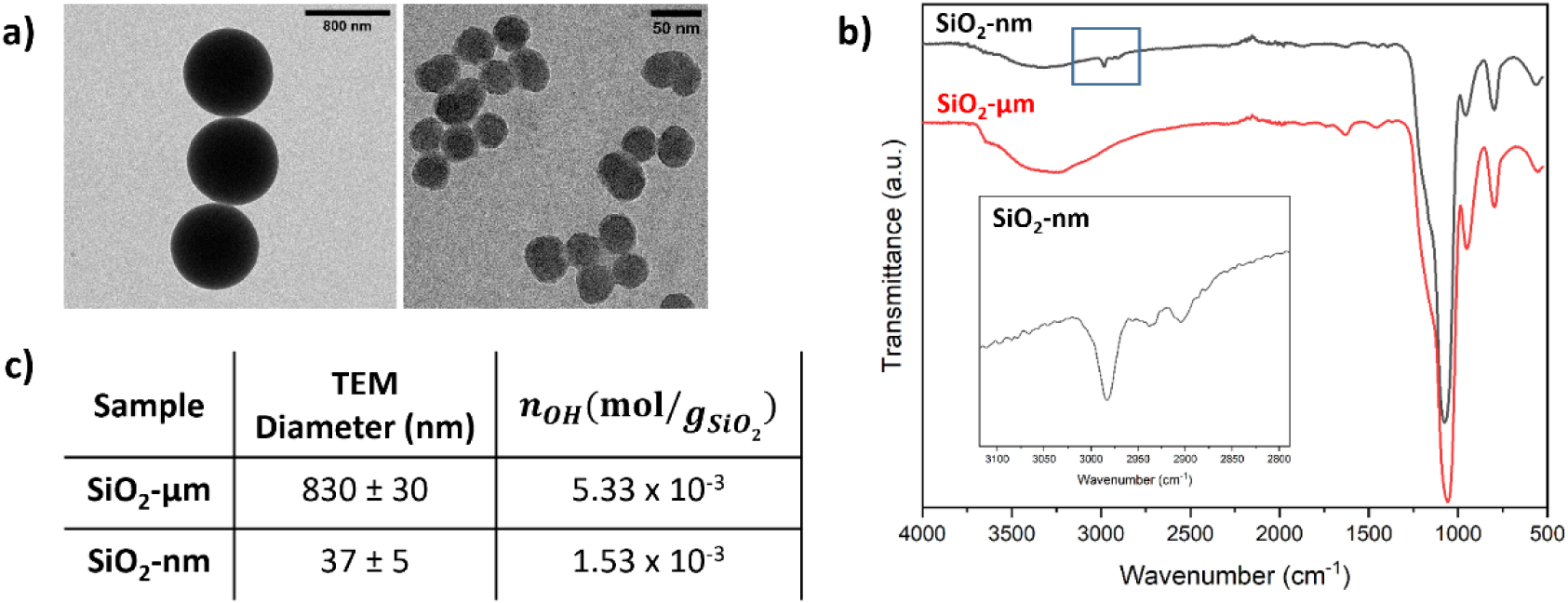
Characterization of SiO_2_-μm and SiO_2_-nm particles: (a) TEM micrographs, (b) ATR-FTIR spectra, and (c) diameter measured by TEM and silanol group quantification via TGA and CHNS analyses.

SiO_2_-μm and SiO_2_-nm present a diameter of 830 ± 30 nm and 37 ± 5 nm, respectively (Figure 1a), showing good monodispersity. From the FTIR spectra of both SiO_2_-μm and SiO_2_-nm (Figure 1b), the intense absorption peak at 1060–1050 cm^–1^ was associated with the Si–O–Si asymmetric stretching, and the peaks at 950 cm^–1^ and 790 cm^–1^ identified as the Si–OH and Si–O–Si stretching vibrations, respectively. For SiO_2_-nm, the peak due to aliphatic C–H stretching of the residual ethoxy groups is present around 3000–2950 cm^–1^. On the contrary, bare SiO_2_-μm shows a higher level of hydration, as observed at 3000–4000 cm^–1^ by the broad band attributable to the stretching of adsorbed water molecules.^38^

The concentration of surface silanol groups (Figure 1c) was estimated by TGA analysis.

Since, in Pickering emulsions, the particles should be wetted both by the dispersed and the continuous phases^39^ it is necessary to partially hydrophobize silica by the introduction of a surfactant (CTAB) capable of increasing the affinity of silica toward paraffin wax.

The concentration of the surfactant must not be too high to avoid the complete embedding of particles in the paraffin wax, as well as not too low in order to promote the particles’ adsorption. Its optimal concentration was determined by measuring ζ-potential values of both SiO_2_-μm and SiO_2_-nm in H_2_O dispersions at different CTAB/SiO_2_ ratios (Figure 2).

**Figure 2:**
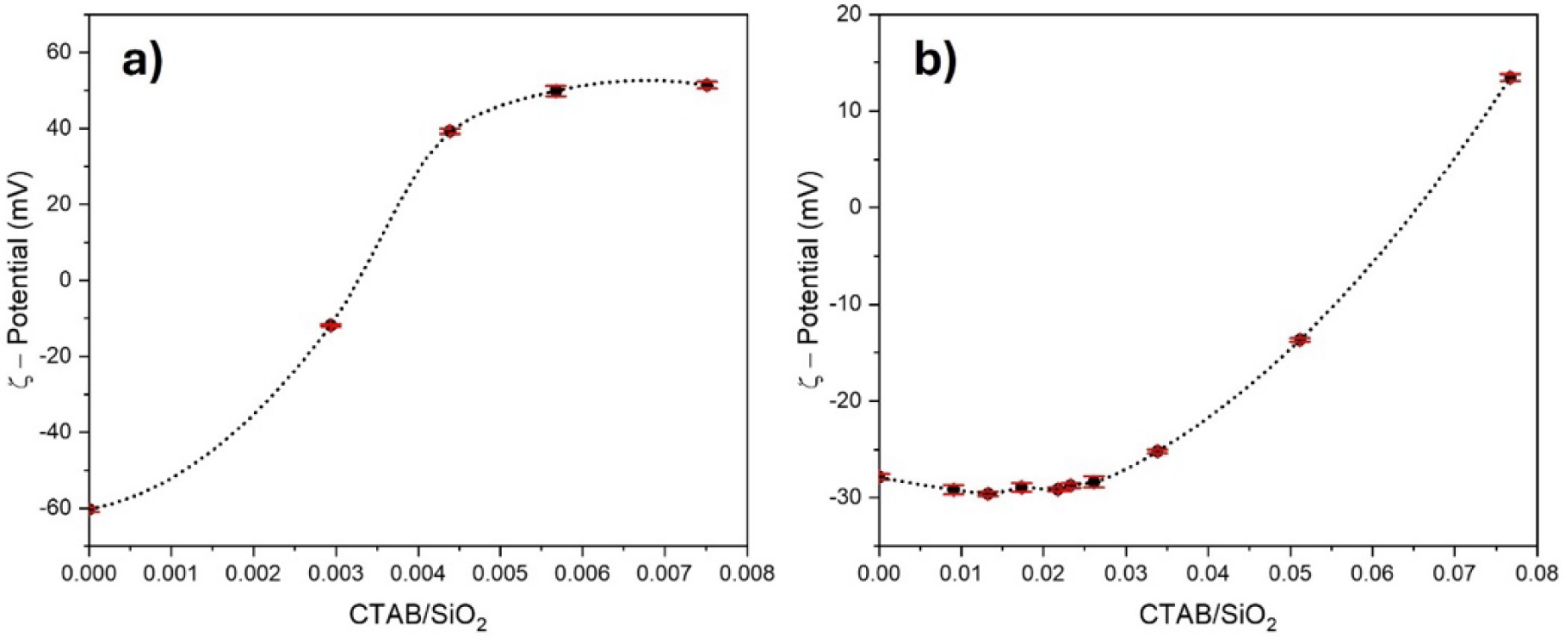
ζ-potential values at varying CTAB/SiO_2_ weight ratios for both (a) SiO_2_-μm and (b) SiO_2_-nm particles. The error bars are highlighted in red.

As expected for colloidal silica particles, both silicas have negative ζ-potential values in the absence of CTAB at pH 7.^40^ With an increase in the CTAB content, the SiO_2_ ζ-potential approaches 0 mV and then becomes positive. The preparation of Pickering emulsions was investigated at approximately 0 mV, with the SiO_2_ particles being significantly hydrophobized by the surfactants and having very small surface charges, which could not hinder the particles’ packing on the wax spheres.

The appropriate concentration of surfactant was determined from the interpolation of the measured ζ-potential values, calculating the CTAB/SiO_2_ weight ratio at 0 mV: 3.285 × 10^–3^ and 6.578 × 10^–2^ respectively for SiO_2_-μm and SiO_2_-nm. The CTAB concentration necessary to compatibilize SiO_2_ particles with paraffin wax is higher in the case of SiO_2_-nm compared to that of SiO_2_-μm, coherently with the higher surface area.

### 3.2 Pickering Emulsions Procedure

Once the optimal amount of CTAB to add is established, above a certain threshold, it is possible to obtain a Pickering emulsion regardless of the specific quantity of paraffin wax used. However, in order to guarantee (i) optimized material quantities for scale-ups and (ii) a good distribution of the particles on the wax colloidosome for the subsequent selective functionalization, the best paraffin wax/silica quantity ratio must be defined. Once these parameters were optimized at the microscale, the same conditions were transferred to the nanoscale system. This two-step approach allowed us to (i) validate the robustness of the protocol and (ii) ensure that the optimized conditions would reliably yield Janus particles with nanometric dimensions.

To investigate the possibility of combining different quantities of silica and paraffin wax, the Pickering emulsions were prepared in the presence of fixed concentrations of CTAB with 0.1 g of SiO_2_-μm by varying the paraffin wax quantity, and they were later analyzed by OM. As reported in Figure 3, the resultant Pickering emulsion obtained with SiO_2_-μm shows poor quality, with higher irregularity in the morphology of the wax colloidosomes (brown circles) and greater agglomeration of silica particles and wax at increasing silica concentrations.

**Figure 3:**
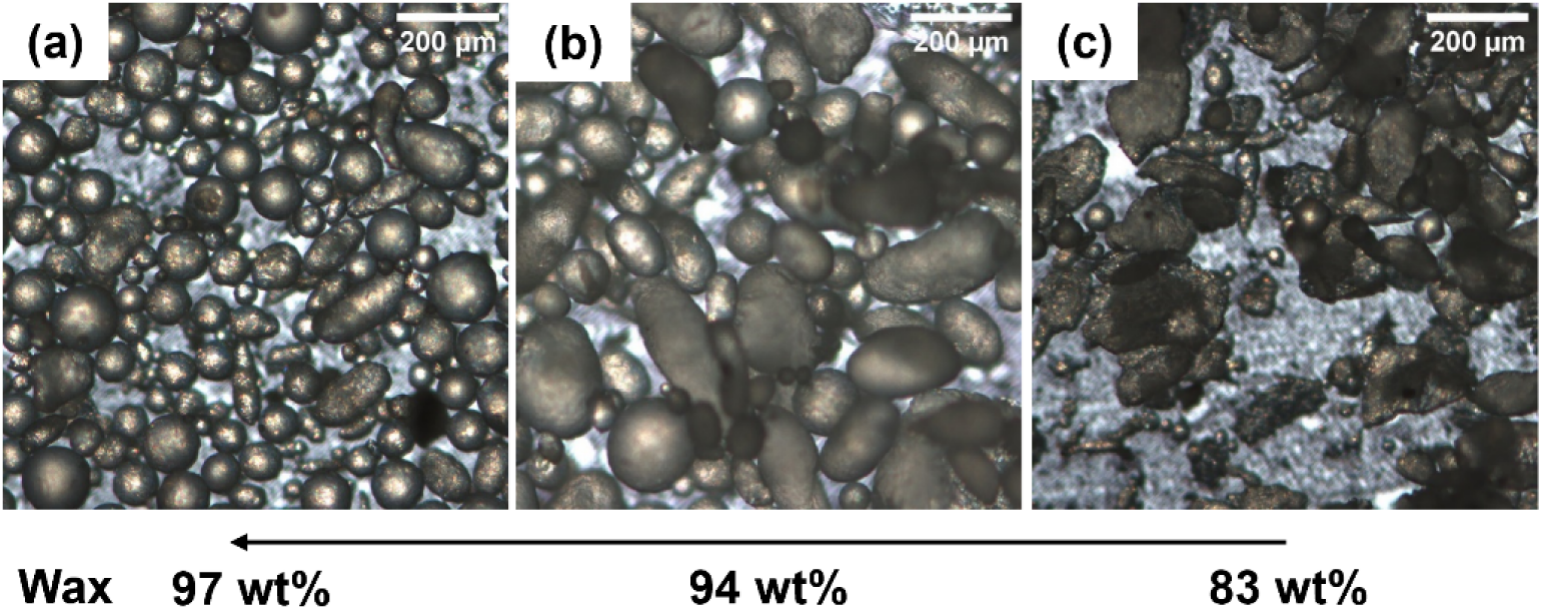
OM images of the colloidosomes resulting from the emulsification process at 97 wt % (a), 94 wt % (b), and 83 wt % (c) of paraffin wax with respect to the total mass of silica and wax in the presence of CTAB after 2 h.

The appropriate combination of silica and wax seems to be around 3 wt % of SiO_2_-μm, in agreement with the data reported by the Granick group^33^ for silica particles having a diameter of 500 nm.

This is related to the fact that by optimizing the silica/wax ratio, a more homogeneous emulsion is obtained. On the contrary, at higher SiO_2_-μm concentrations, the quantity of wax is not enough to guarantee the formation of emulsion-like spherical colloidosomes characterized by an optimal distribution of the particles at their interface with water.

It must be considered that, as generally observed for emulsion preparations, at different emulsification times, the Pickering emulsion changes in terms of colloidosome homogeneity. Therefore, the evolution of the Pickering emulsion in terms of colloidosome diameters and PDI was investigated at different times by OM analysis (Figure 4 and Table 2).

**Table 2:** The Measured Diameters and PDI Values of the Colloidosomes at 3 wt % of SiO_2_-μm at Different Emulsification Times

Time (min)	15	30	45	60	120
Diameter (μm)^a^	112 ± 49	103 ± 75	63 ± 30	87 ± 48	79 ± 25
**PDI**	0.66	0.85	0.69	0.74	0.57

a The reported diameters are an average of 50 colloidosomes.

**Figure 4:**
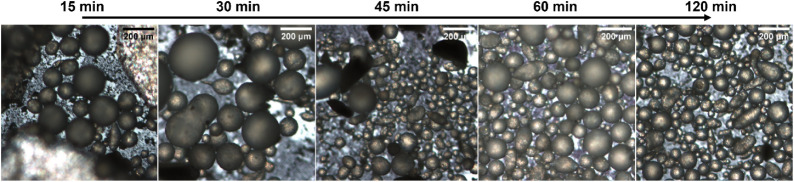
OM images of the colloidosomes prepared by the emulsification process between SiO_2_-μm (3 wt %) and paraffin wax in the presence of CTAB at different times. In the first image, residual pieces of wax not included in the emulsion are evident (white area).

After 2 h from the beginning of the emulsification process, the colloidosomes become more monodisperse, with a substantial reduction in the average diameter compared to the first 15–30 min of emulsification. A digital photo of the formation of the Pickering emulsion is reported in Figure S1 to provide a clear, macroscopic view of the system. These images complement the SEM and TEM observations, allowing readers to correlate the bulk appearance of the emulsion with the microscopic structures of the particles and the stabilized droplets.

In detail, the colloidosomes are very large, and there is a significant amount of nonincorporated silica particles visible as a layer in the background during the first 15–30 min. After 45 min, colloidosomes of smaller dimensions appear, but the system is inhomogeneous. Only after 60 and 120 min does the polydispersity of the system decrease. In addition, at 120 min, a complete coverage of the colloidosomes by silica particles is observed (Figure 5), where silica forms a single layer on the paraffin wax spheres (Figure 5b, c and d), even if some colloidosomes appear irregular in their shape (Figure 5a).

**Figure 5:**
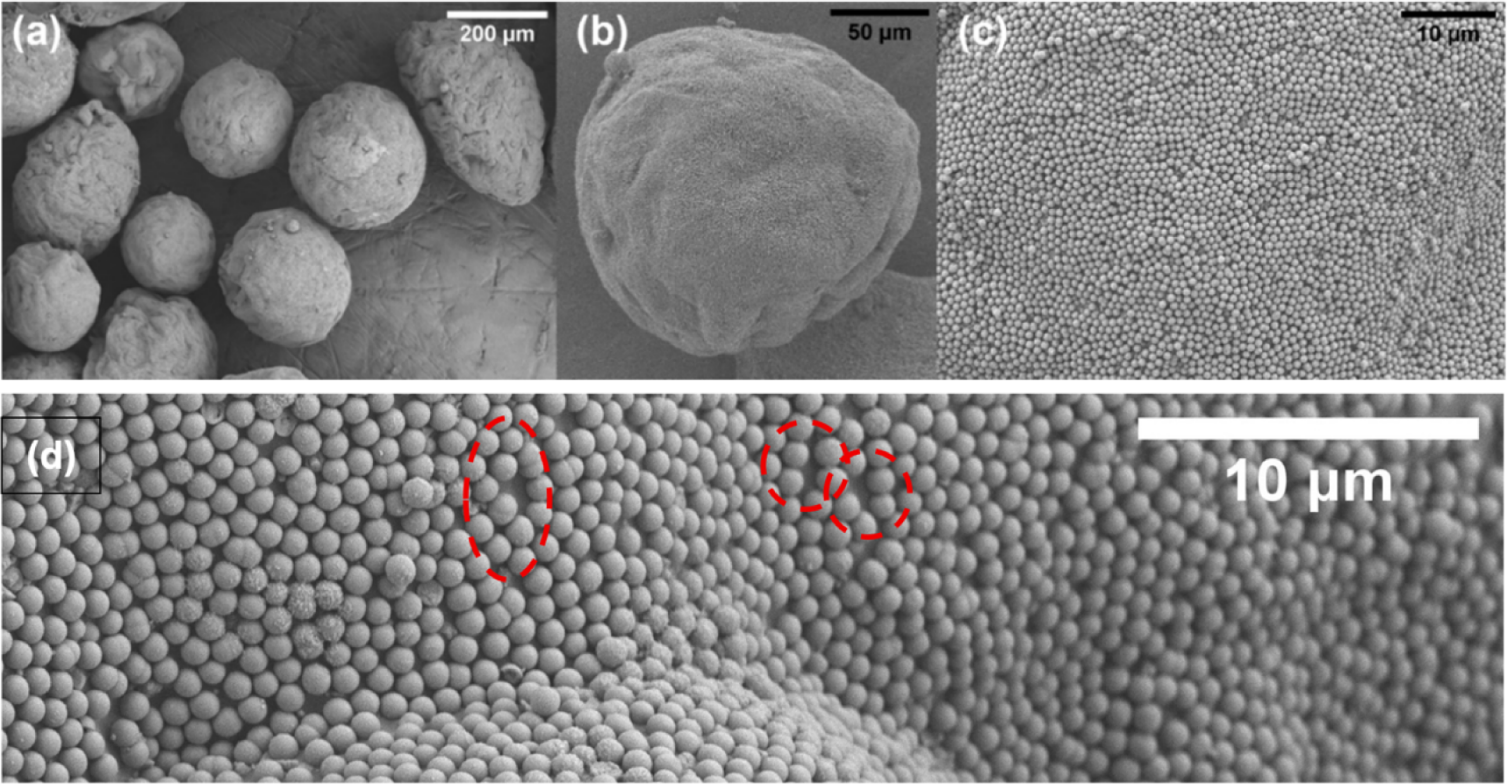
SEM images at increasing magnifications (from a to d) of the colloidosomes resulting from the emulsification process between SiO_2_-μm (3 wt %) and paraffin wax in the presence of CTAB after 2 h. The red circles in (d) highlight the single layer of particles.

These changes over time can be associated with different stages required for the Pickering emulsion to form: first of all, the time required for the paraffin wax to melt, and second, the time required for the emulsion to stabilize and reduce droplet coalescence. In any case, even after extended emulsification times, the colloidosomes remain structurally intact and stable without observable aggregation or collapse.

The silica-to-wax weight ratio was determined by TGA of the colloidosomes (TGA of three representative batches of colloidosomes is reported as an example in Figure S2a) in order to estimate the amount of silica particles that can be functionalized, assuming that all the silica particles present in the colloidosomes are located at the surface. In detail, by the weight loss between 150 and 1000 °C (about 95.45%), attributable to the wax, the surface hydroxyl and ethoxy groups of silica, and CTAB, the quantities of wax burnt as CO_2_ and of silica as the remaining residue at 1000 °C were determined. SiO_2_-μm/wax ratio was obtained as the average of three repetitions: a good correlation exists between the measured SiO_2_-μm/wax weight ratio (0.035 value by TGA) and the theoretical one (0.033, determined by considering the amount of silica and wax introduced in the emulsification environment), in line with that reported by Granick et al.^33^ for silica particles of 500 nm.

Once the effectiveness of this approach was determined for the preparation of Pickering emulsions of micrometric silica and paraffin wax, the same method was applied for the preparation of the emulsions with SiO_2_ at the nanoscale (see Paragraph S2 for further details). The CTAB concentration was adjusted, taking into account the different surface characteristics of the SiO_2_-nm NPs, while the most appropriate contents of SiO_2_-nm and wax were determined by TGA of Pickering emulsion colloidosomes prepared at different SiO_2_-nm/wax ratios (TGA of three representative batches of colloidosomes is reported as an example in Figure S2b). Interestingly, compared to SiO_2_-μm/wax Pickering emulsions, a high amount of nonincorporated wax is present as a superficial layer after the emulsification is stopped. As a result, regardless of the added wax quantity, the colloidosome TGA always results in a nominal SiO_2_-nm/wax weight ratio of 0.159, significantly shifted from the value of 0.033 obtained for SiO_2_-μm.

The evolution of the emulsion morphology, as well as the particle organization as a function of time (15–120 min) at a fixed SiO_2_/wax value equal to 0.159, was studied using OM and SEM analyses (Figures 6,7). After 15 min (Figure 6), the SiO_2_-nm emulsion shows the presence of large wax spheres together with undefined and aggregated mixtures of silica and wax. However, after 2 h, these aggregates seem to be replaced with small and defined colloidosomes. In fact, after 2 h, the PDI value (Table 3) is higher compared to the values measured for the emulsion prepared with SiO_2_-μm (Table 2), due to the presence of both large and small colloidosomes (Figure S3). Compared to the Pickering emulsion prepared at higher wax contents (SiO_2_/wax = 0.033, Figure S4), it seems that at lower paraffin wax contents (SiO_2_/wax = 0.159), for the silica particles to be incorporated on the surface of the wax colloidosomes, a longer mixing time is required.

**Table 3:** The Measured Diameters and PDI Values of the Colloidosomes of SiO_2_-nm at Different Emulsification Times

Time (min)	15	30	45	60	120
Diameter (μm)^a^	108 ± 58	97 ± 64	112 ± 70	109 ± 48	71 ± 48
**PDI**	0.73	0.81	0.79	0.67	0.82

a The reported diameters are an average of 80 colloidosomes.

**Figure 6:**
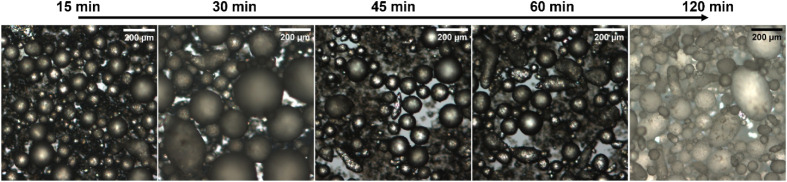
OM images of the colloidosomes prepared by the emulsification process between SiO_2_-nm and paraffin wax at a SiO_2_/wax ratio of 0.159 in the presence of CTAB at different times.

**Figure 7:**
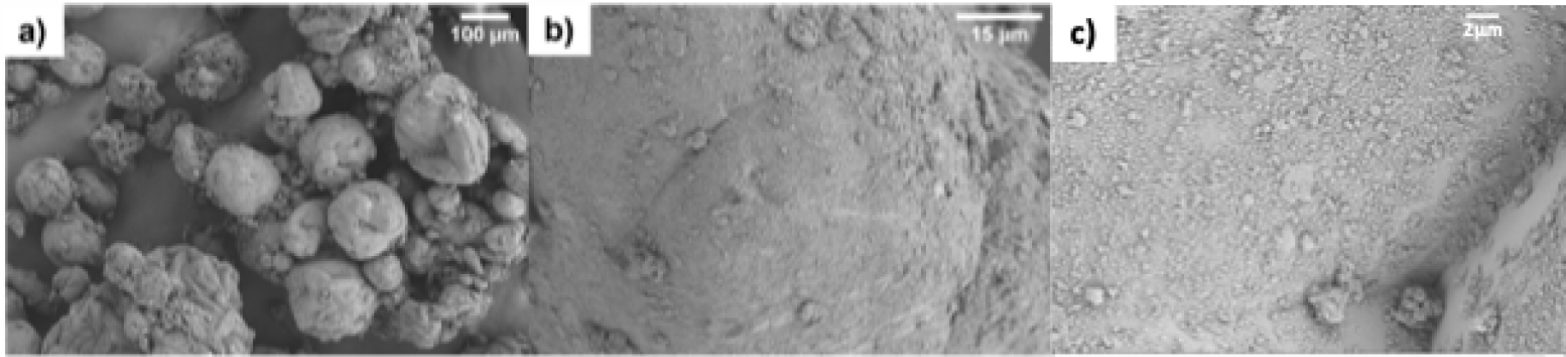
SEM images at lower (a) and higher (b, c) magnifications of the colloidosomes prepared by the emulsification process between SiO_2_-nm and paraffin wax in the presence of CTAB at a SiO_2_/wax ratio of 0.159.

The higher polydispersity should not represent a drawback for subsequent steps, as it is more decisive for achieving homogeneous surface coverage by silica particles. This is certainly confirmed by SEM images (Figure 7) where a homogeneous coverage of the colloidosomes is evident without significant overlapping of layers of silica particles, which is significantly different from what we observed for the emulsion prepared with higher paraffin wax quantities (Figure S6).

Therefore, once the correct amount of CTAB is defined, it appears possible to further optimize the SiO_2_/wax ratio just by performing TGA of the retrieved colloidosomes (Figure S2). It must be noted that the colloidosomes resulting from an unoptimized emulsion (SiO_2_-nm/wax ratio of 0.033) consist of overlapping layers of silica particles and wax, which are unfavorable for the subsequent functionalization step, and possess high polydispersity even if improved with increased mixing time (Table S4). However, the correct silica–paraffin wax colloidosome combination seems to be retained regardless of the quantities introduced in the emulsification environment. This could be associated with the fact that the most important parameter to adjust in order to guarantee the formation of the emulsion is the amount of CTAB, which in turn affects the particles’ surface charge and hydrophobicity. In any case, in the presence of an excess of wax, only the content required for emulsion formation will be incorporated and stabilized by silica NPs.

To scale up the procedure with larger amounts of both micrometric and nanometric silica, the Pickering emulsion was prepared at increasing quantities (incorporating 0.2 and 0.4 g of SiO_2_ particles) while maintaining the same SiO_2_/wax and CTAB/SiO_2_ ratios. Even though an increase in the irregularities of the morphology and size of the colloidosomes occurs, along with the presence of a significant amount of smaller wax spheres (Figures S3 and S4), the colloidosome coverage by both SiO_2_-μm and SiO_2_-nm remains good. Therefore, it appears that, by following the determined CTAB concentration and SiO_2_/wax ratio, the combination was optimized to a level at which it is possible to achieve complete particle adsorption and thus scale up the procedure without further tuning the parameters.

Following these results, the optimized SiO_2_-μm and SiO_2_-nm Pickering emulsions were considered for the subsequent functionalization steps.

### 3.3 Selective Functionalization of SiO_2_ Particles: APTES-Functionalized JNPs

To better investigate the optimal conditions for the selective functionalization of silica particles adsorbed on the surface of paraffin wax spheres, the stability of the colloidosomes in solutions of EtOH and H_2_O at different compositions was studied. In fact, solvents able to dissolve or partially damage paraffin wax should be avoided to preserve the colloidosome integrity.

Interestingly, after dispersing the colloidosomes in EtOH, a drastic change in the surface coverage by silica particles was observed for both silicas, independent of their dimensions (Figures S7 and S8). In detail, the colloidosomes appear partially intact, but most of the silica particle layers have been detached and significantly damaged in the case of SiO_2_-μm.

Being, therefore, impossible to functionalize selectively silica with APTES in EtOH or an organic solvent able to dissolve paraffin wax, the colloidosomes’ stability was investigated at different EtOH/H_2_O contents. Up to a 50/50 EtOH/H_2_O ratio (i.e., 50/50 and 30/70 ratios in Figures 8 and S7), the colloidosomes appeared intact without any significant variation in their structure and coverage by silica particles.

**Figure 8:**
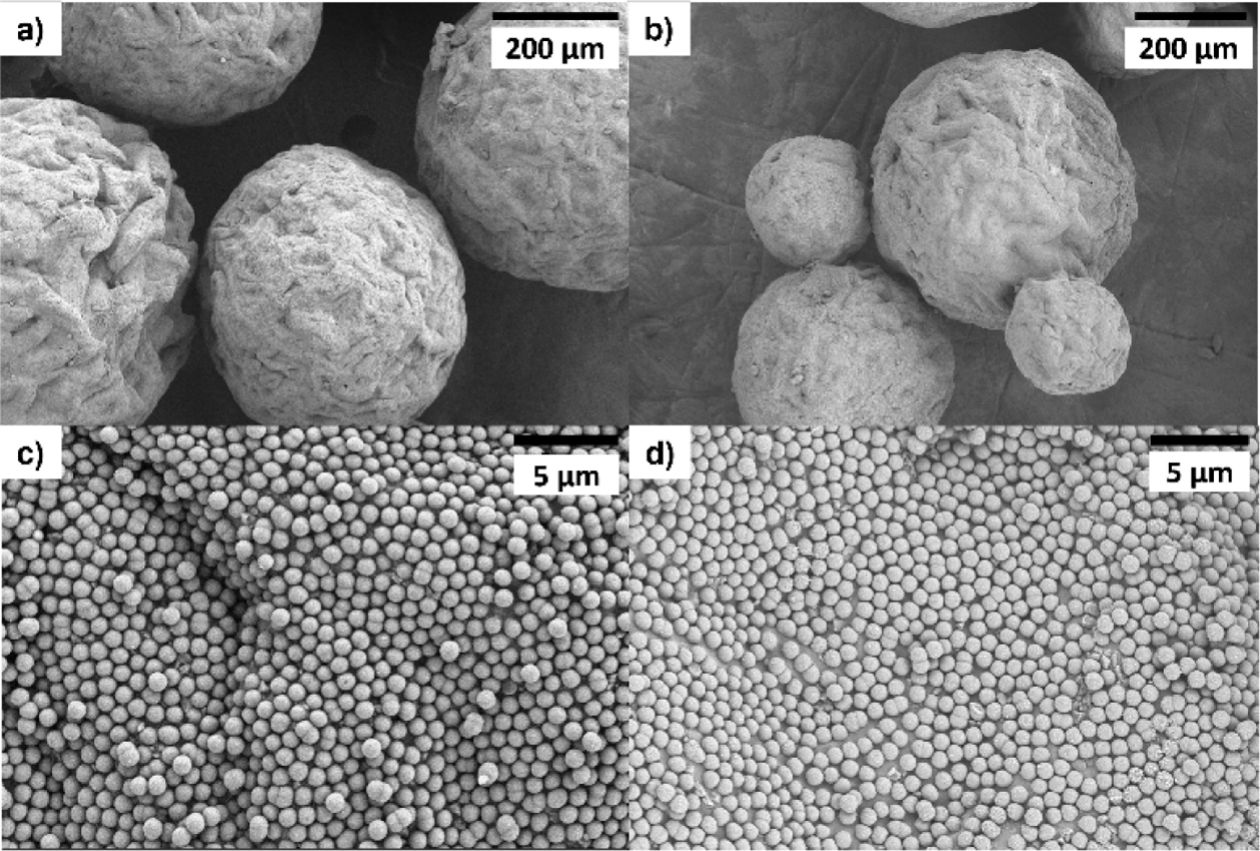
SEM images of the SiO_2_-μm/wax colloidosomes prepared at a 0.033 SiO_2_/wax ratio after being redispersed in EtOH/H_2_O 50/50 (a, c) and 30/70 (b, d) at different magnifications.

Starting from these considerations, the functionalization of silica was investigated in a mixture of EtOH and H_2_O (50/50) in order to keep the integrity of the colloidosomes but, at the same time, avoid high H_2_O concentrations that could lead to the self-condensation of the silane.^41^ Besides, the reaction temperature was kept lower than 50 °C due to the low melting temperature of paraffin wax (53–58 °C), in order to avoid any possible rotation of the particles on the colloidosomes’ surface.

Given the possibility that the presence of CTAB hinders the functionalization of silica particles, the emulsions were prepared at a CTAB concentration corresponding to a ζ-potential of approximately −10 mV (data not reported). Under these experimental conditions, worse colloidosome coverages were observed, and thus, the functionalization with APTES was performed directly on the emulsions prepared by targeting a CTAB concentration corresponding to a ζ-potential of 0 mV.

In detail, once an optimal Pickering emulsion was achieved for SiO_2_-nm, the functionalization step was conducted preliminarily for SiO_2_-μm (Paragraph S4) and subsequently deeply investigated for SiO_2_-nm particles, which are more suitable for the final application than SiO_2_-μm.

The functionalization reaction was first set up on bare SiO_2_-nm particles (Paragraphs S4 and S5) and then on silica/wax colloidosomes. Concerning the APTES concentration, the silane was added in excess compared to the estimated *n_OH_* (mol/*g*_SiO2_) value but in a concentration based on an APTES: surface OH groups of SiO_2_-μm ratio equal to 1:1. In this way, the variability associated with the mild functionalization conditions and the fact that, in most reaction environments, the ethoxy groups are partially hydrolyzed is limited.

The APTES-JNPs were characterized by TGA and CHNS after paraffin wax removal in toluene to determine the amount of grafted silane molecules.

With respect to wax residues, TGA analysis (Figure S10) shows that the weight loss of APTES-JNPs is comparable to that of fully APTES-functionalized silica particles, thus excluding the presence of wax. The thermograms are particularly informative in the temperature range between 200 and 300 °C, where wax degradation is expected (Figure S2). In this region, no significant differences are observed between the fully functionalized sample (green curve, SiO_2_-nm APTES) and the JNPs (red and blue curves). Although the curves are slightly shifted due to the initial water loss, their overall weight-loss trends are essentially identical. This close correspondence confirms that any residual wax is completely removed from the APTES-JNP samples during the toluene washing step.

The quantity of APTES reached by functionalizing the adsorbed silica is about 1.52 wt %, in line with the 1.76 wt % value obtained by CHNS analysis (Table S9), and comparable to the one obtained for the completely APTES-functionalized SiO_2_ NPs (Table S6). This result could seem contradictory: if the particles are partially embedded in paraffin wax, then a smaller surface area should be available for functionalization. However, it is possible that the silica NPs employed for the preparation of Pickering emulsions are more exposed to subsequent hydrolysis reactions of the ethoxy groups. As a result, adsorbed NPs have different surface properties that can modify the reactivity of the available surface area. At the same time, the colloidosomes could have a different solvent dispersion ability compared to bare SiO_2_ NPs, with consequent changes in the amount of available surface area.

The effective localization of the functionalizing agent was confirmed by dispersing APTES-JNPs in a suspension of Au NPs in EtOH/H_2_O. The solvent composition (40/60 v/v) was selected so that the Au NPs could locate on the SiO_2_ surface without forming aggregates, thus leading to the formation of a single layer of gold spheres.^42^ In this way, their spontaneous assembly around the grafted surface area presenting −NH_2_ functionalities could confirm the selective localization of the Au NPs. The same procedure was applied to the completely APTES-functionalized SiO_2_-nm sample to evidence, in this case, the random anchorage of Au NPs to the whole surface of silica NPs. As confirmed by TEM analysis (Figure 9), in the case of SiO_2_ APTES-JNPs, the Au NPs are localized in specific areas of the SiO_2_ NP surface, thus confirming their Janus nature (a in Figure 9 and Figure S11). On the contrary, when the Au NPs are dispersed on completely APTES-functionalized NPs, their localization seems to be random (Figure 9b).

**Figure 9:**
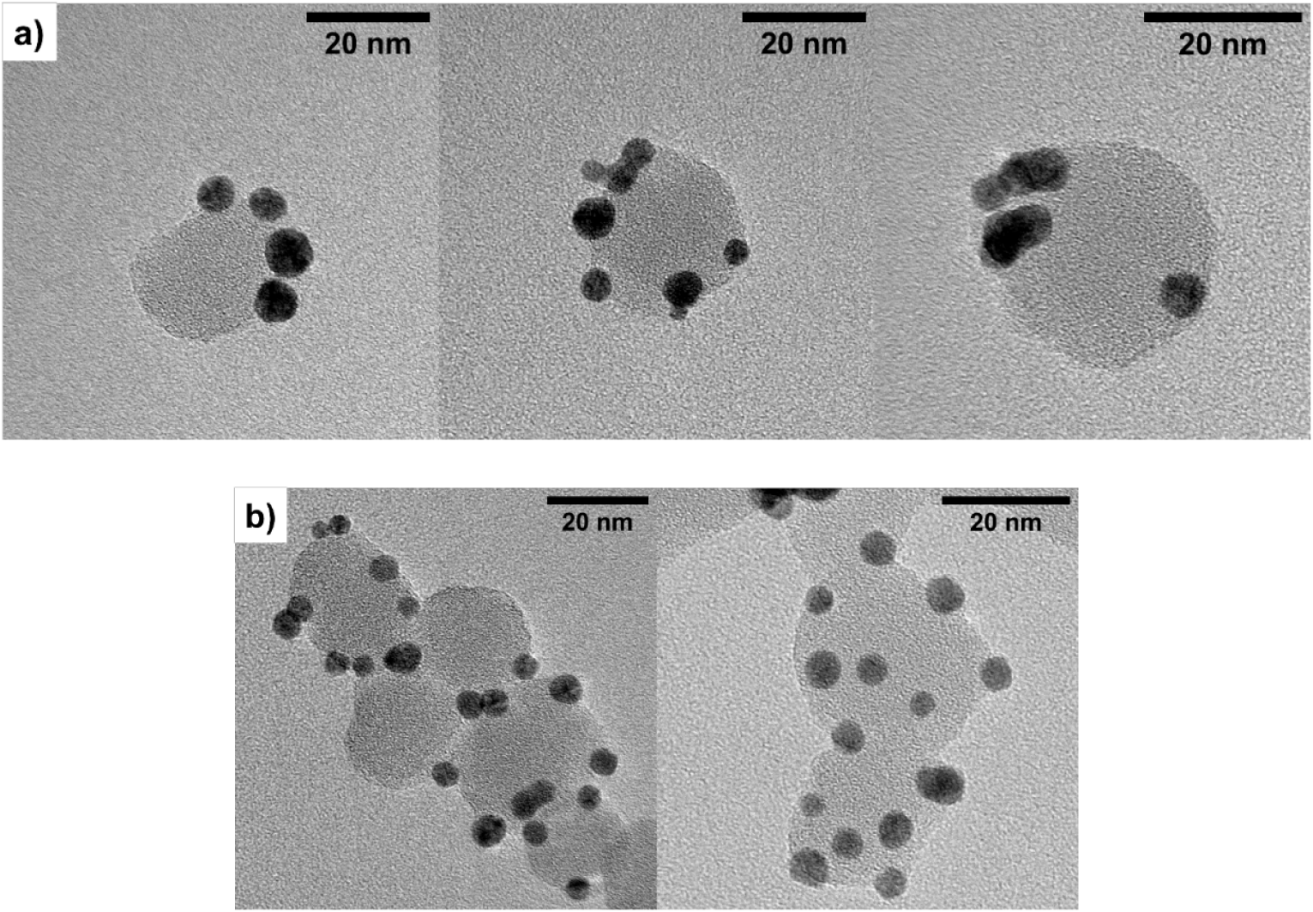
TEM micrographs of the Janus heterodimer with Au NPs localized only on one hemisphere of APTES-JNPs (a) and the heterodimer with Au NPs randomly dispersed on the SiO_2_-nm NPs surface (b).

### 3.4 PS- and PB-Grafted Janus NPs

With the aim of obtaining amphiphilic JNPs of different natures, APTES-JNPs were modified by grafting different kinds of polymer chains, such as PB and PS, which have low and high glass transition temperatures and thus different applications. In detail, commercially available PB and PS oligomers with maleic anhydride functionalities were grafted by exploiting the APTES amino groups present on SiO_2_ APTES-JNPs (Scheme 2).

**Scheme 2:**
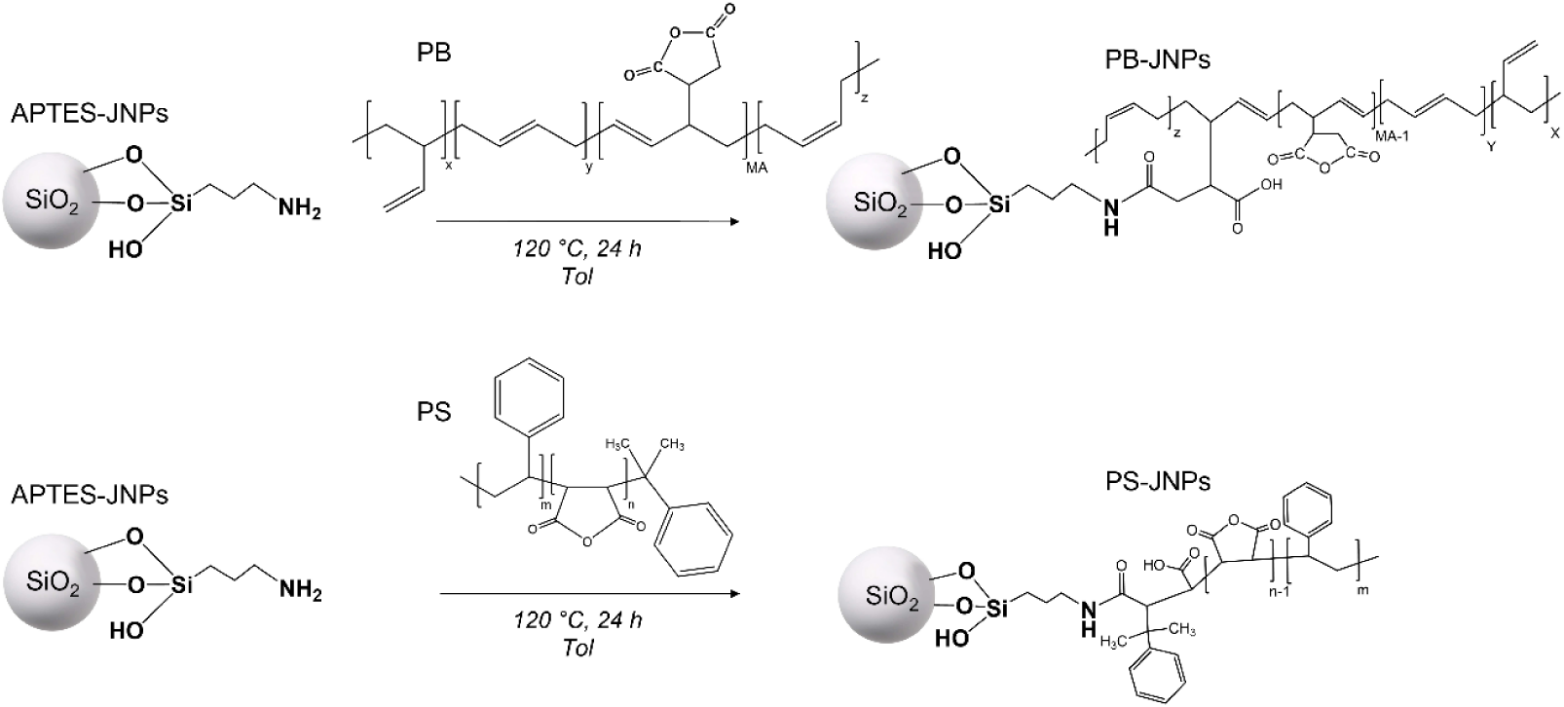
The Preparation of PS- and PB-Grafted Janus NPs

The quantification of the grafted polymer chains was performed by TGA analysis (Table S10) which resulted in 14.11 and 12.12 wt % for PS and PB, respectively. A higher PS amount was grafted thanks to its shorter chains compared to PB and thus a lower steric constraint. The spectroscopic characterization (not reported) confirms the presence of the grafted polymer, in agreement with.^6^

Furthermore, to confirm the selective grafting of the oligomer’s chains on SiO_2_ APTES-JNPs, the localization of PS-JNPs in toluene and H_2_O (50/50) dispersions was analyzed. As shown in Figure 10, just after sonication and vigorous shaking of the dispersion, the polymer-grafted JNPs significantly migrated from the aqueous phase at the bottom to the interface between toluene and H_2_O. Their amphiphilic Janus nature can thus be confirmed by their spontaneous migration.

**Figure 10:**
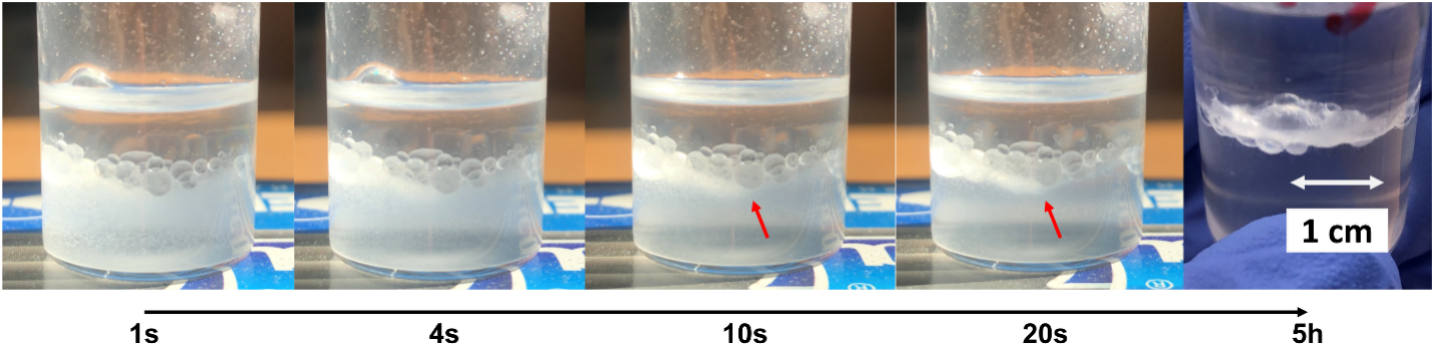
Pictures of a toluene-H_2_O dispersion of PS-JNPs at different times after sonication and vigorous shaking. Already after 10–20 s, the PS-JNP migration at the interface is confirmed by the formation of a white line highlighted by the red arrows. After 5 h, the same line is particularly evident at the interface between the organic and aqueous phases.

In addition, in the first seconds, a partial reduction in the volume of the upper phase (toluene) compared to the aqueous phase was distinguishable at the bottom, alongside the formation of bubbles. It is therefore possible to hypothesize that these bubbles consist of toluene molecules dispersed in the aqueous phase and stabilized by the presence of PS-JNPs at the interphase. The amphiphilic nature of PS-JNPs was further confirmed after 5 h (after the disappearance of the bubbles at the interface), when a particularly defined line composed of SiO_2_ PS-JNPs formed at the interphase.

## 4 Conclusions

A procedure for the preparation of SiO_2_-paraffin wax Pickering emulsions was established, starting from an in-depth analysis of Granick’s method. By following the defined guidelines, it is now possible to find the best conditions for the formation of Pickering emulsions, starting from particles of different natures, consistent with their surface chemistry and dimensions. At the same time, in this way, the synthesis of JNPs by Pickering emulsions can now be extended to particles with dimensions smaller than 100 nm. This inventive step will extend the application of JNPs to a wider range of nanocomposites and hybrid materials, for which nanometric particle size is a mandatory requirement.

The selectively modified SiO_2_ APTES-, PS-, and PB-JNPs show specific behaviors, confirming their dipolar and amphiphilic nature, respectively. In detail, the selective modification with APTES was confirmed by the adsorption of Au NPs, while the NPs’ spontaneous assembly at an aqueous–organic interphase demonstrated the selective anchoring of PS and PB oligomers.

In addition, even though many constraints in the SiO_2_ NPs functionalization have been identified (limiting temperatures, EtOH/H_2_O mixtures, half of the surface availability), a satisfactory APTES wt % of approximately 1.5 wt % has been achieved even under very mild conditions. Furthermore, starting from these APTES percentages, we have also confirmed the possibility of achieving high polymer grafting.

The resultant particles show promising surfactant behavior, which could find applications in different contexts, starting from Pickering emulsion stabilization and extending to polymer blend compatibility improvements. In addition, the as-prepared PS- and PB-JNPs could be further modified, thus obtaining additional building blocks depending on the targeted material.

## Supplementary Material



## Data Availability

Data will be made available upon request.

## Notes

Synthesis and characterization of silica NPs; Synthesis and characterization of silica/wax colloidosomes: *Digital photo of the formation of Pickering emulsion; TGA analysis of SiO*_2_ *-μm/wax; SiO*_2_ *-μm/wax colloidosomes, SiO*_2_ *-nm/wax colloidosomes*; Study of SiO_2_ colloidosomes in ethanol/water mixture; Functionalization of silica particles with APTES; Functionalization of silica colloidosomes with APTES. Au-JNPs heterodimer; PB and PS grafting of APTES JNPs; PB and PS grafting of APTES JNPs (PDF)
