# Supplementary material for: Janus Nanoparticles by Pickering Emulsions: A Versatile Approach for the Selective Functionalization of SiO2
Source: ACS Appl Mater Interfaces. 2026 Jan 28;18(5):9043–53. doi: 10.1021/acsami.5c20303 (PMC12903102; doi:10.1021/acsami.5c20303)
Supplement: Supplementary file 1 [file am5c20303_si_001.pdf]

Supporting Information

Janus Nanoparticles by Pickering Emulsions: a versatile approach for the selective functionalization of SiO_2

Elisa Manzini,[a] Silvia Mostoni,[a] Massimiliano D'Arienzo,[a] Luciano Tadiello,[b] Roberto Scotti,[a] and Barbara Di Credico[a,*]

[a]*Department of Materials Science, INSTM, University of Milano-Bicocca, Via Roberto Cozzi, 55, 20125 Milan, Italy; *barbara.dicredico@unimib.it*

[b]*Pirelli Tyre S.p.A., Viale Piero e Alberto Pirelli, 25, 20126 Milan, Italy*

S1. Synthesis and characterization of silica NPs

The dimensions of both SiO_2-µm and SiO_2-nm, measured by DLS and TEM analyses, are reported in Table S1. At increasing reaction temperatures and decreasing TEOS and H_2O concentrations, silica particles size decreases significantly.

Table S1 – SiO_2 particles diameters.

Sample	DLS hydrodynamic diameter[a] (nm)		TEM diameter[a] (nm)
	Number	Intensity	
SiO_2-µm	896 ± 193	940 ± 178	830 ± 30
SiO_2-nm	43 ± 15	146 ± 108	37 ± 5

[a] The reported diameter is the number average of at least six subsequent measurements; the uncertainty is the standard deviation.

In addition, the BET analysis (Table S2) confirms the significantly smaller surface area per gram of SiO_2-µm, correlated to a larger particle size.

Table S2 – SSA and particle size of SiO_2 particles.

Sample	SSA (m²/g)	Particle size (nm)
SiO_2-µm	3.748	1531.19
SiO_2-nm	181.6	31.87

SiO_2 particles were further characterized by TGA and CHNS analyses in order to determine the amount of silanols (-OH) and ethoxy groups (EtO-) present on the particles surface. These values are significantly important for the determination of the available reacting hydroxyl groups present on silica for the subsequent functionalization step. From the silica thermograms it is possible to identify two weight losses associated to: i) the elimination of the solvent molecules adsorbed on silica NPs (30 – 150 °C) and ii) of the –OH groups and the non-hydrolysed –OEt groups as H_2O and CO_2 molecules (150 – 1000 °C) respectively.

$$\Delta \mathrm{wt}\%_{150-1000\ °C}^{SiO_2} = wt\%_{OH+OEt} \quad \text{(Eq. S1)}$$

where:

- $\Delta \mathrm{wt}\%_{150-1000\ °C}^{SiO_2}$ is the sample weight loss between 150 and 1000 °C;
- $wt\%_{OH+OEt}$ is the weight percentage of silanol and ethoxy groups.

To exclude from this value the –OEt groups component, their amount was calculated starting from the registered carbon content ($\%C$) by Eq. S2:

$$Wt\%_{OEt} = \frac{MW_{OEt} \cdot \%C}{2 \cdot MW_C} \quad \text{(Eq. S2)}$$

where:

- MW_{OEt} is the molecular weight of ethoxy group;
- MW_C is the molecular weight of carbon.

By combining the information obtained by TGA and CHNS analyses, it is thus possible to estimate the quantity of -OH groups present on the silica surface by Eq. S3 considering that for every two silanol groups, one H_2O molecule is eliminated:

$$n_{OH}(\text{mol}/g_{SiO_2}) = \frac{\Delta \text{wt}_{150-1000\ °C}^{OH} \cdot 2}{MW_{H_2O} \cdot w_{1000\ °C}} \quad \text{(Eq. S3)}$$

Where:

- $\Delta \text{wt}_{150-1000\ °C}^{OH}$ is the weight loss associated to the -OH groups only;
- MW_{H_2O} is the molecular weight of water;
- $w_{1000\ °C}$ is the weight of the sample at 1000 °C.

The trends in the $wt\%_{OEt}$ and n_{OH} values, reported in Table S3, can be explained considering a higher probability of hydrolysis phenomena in the reaction environment of SiO_2-µm due to the presence of a higher H_2O concentration. However, the amount of hydroxyl groups does not differ significantly from the one registered for SiO_2-nm because of the higher surface area per grams of SiO_2 NPs.

Table S3 – The wt% of carbon, the silanols and ethoxy weight percentages, and the mole values of silanols for gram of silica particles calculated from Eq. S3. All the reported values are an average of two batches.

**Sample**	$wt\%_{OH+OEt}$ *	**C%**[§]	$wt\%_{OEt}$ *	$n_{OH}(\text{mol}/g_{SiO_2})$ *
SiO_2-µm	5.2 ± 0.8	0.5 ± 0.3	0.9 ± 0.6	5.33 x 10^{-3}
SiO_2-nm	6.6 ± 0.8	2.9 ± 0.7	5 ± 1	1.53 x 10^{-3}

[*]calculated from TGA and CHNS analyses; [§] registered from CHNS analysis

S2. Synthesis and characterization of silica/wax colloidosomes

S2.1 Digital photo of the formation of Pickering emulsion

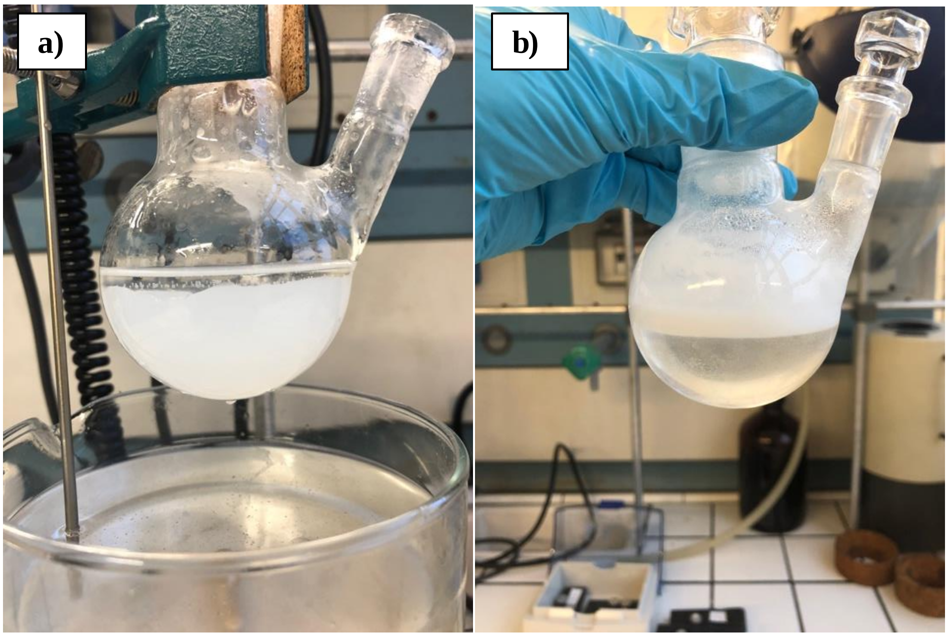

Figure S1. Digital images of the silica dispersion prior to emulsification (a) and after the formation of the Pickering emulsion (b).

S2.2 TGA analysis of SiO_2-µm /wax

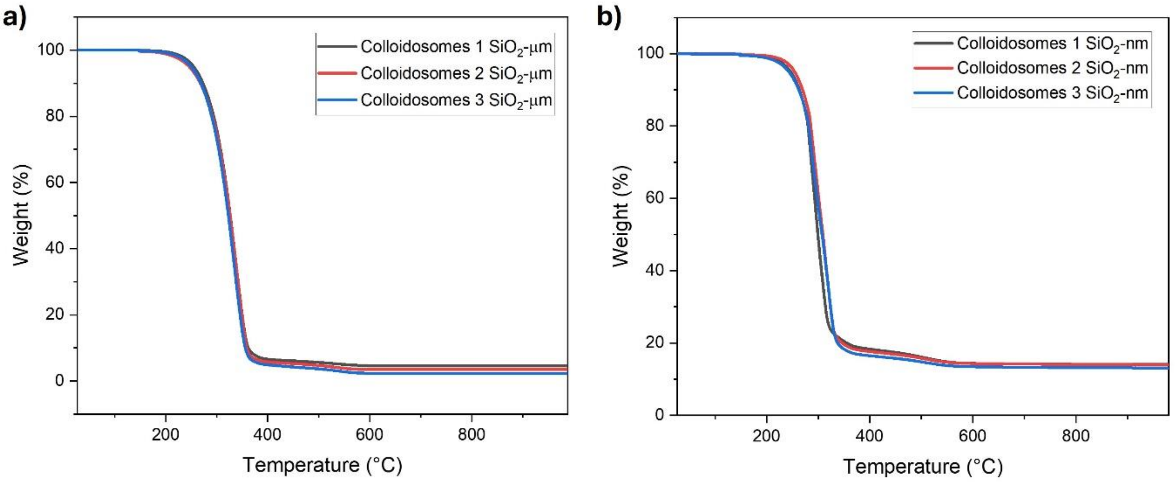

Figure S2. TGA of three batches of colloidosomes reported as an example resulting from the emulsification process between a) SiO_2-µm and paraffin wax and b) SiO_2-nm and paraffin wax

For SiO_2-µm and paraffin (a in Figure), the estimated Δwt % between 150 and 1000 °C was: Colloidosomes 1: 95.45 %, Colloidosomes 2: 96.53 %, Colloidosomes 3: 97.75%.

For SiO_2-nm and paraffin (b in Figure), the estimated Δwt % between 150 and 1000 °C was: Colloidosomes 1: 85.95 %, Colloidosomes 2: 85.98 %, Colloidosomes 3: 86.88%

S2.3 SiO_2-µm /wax colloidosomes

The procedure of the colloidosomes preparation was tested by doubling and quadrupling the quantities, of SiO_2 particles. Figures S3 and S4 a show that the colloidosomes form. At higher magnifications (b in Figures S3 and S4) the coverage by silica particles seems homogeneous with the exception of some areas where the particles seem to have detached from the wax spheres. By analyzing the TEM stub surface it is however evident that no SiO_2 particle is not incorporated in the colloidosomes.

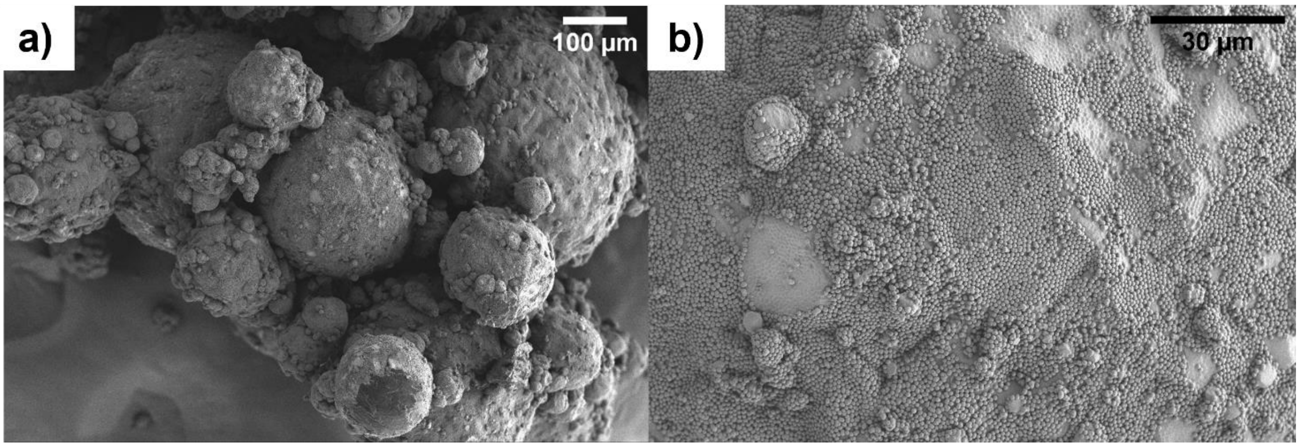

Figure S3 – SEM images of the colloidosomes at different magnification (a, b) resulting from the emulsification process between SiO_2-µm and paraffin wax in the presence of CTAB by doubling the starting materials quantities.

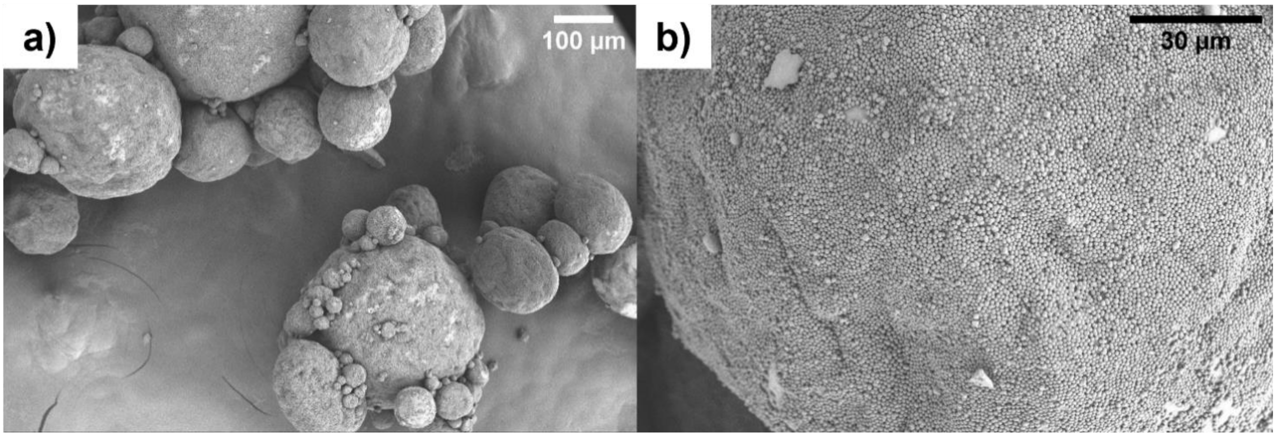

Figure S4 – SEM images of the colloidosomes at different magnification (a, b) resulting from the emulsification process between SiO_2-µm and paraffin wax in the presence of CTAB by quadrupling the starting materials quantities.

S2.4 SiO_2-nm /wax colloidosomes

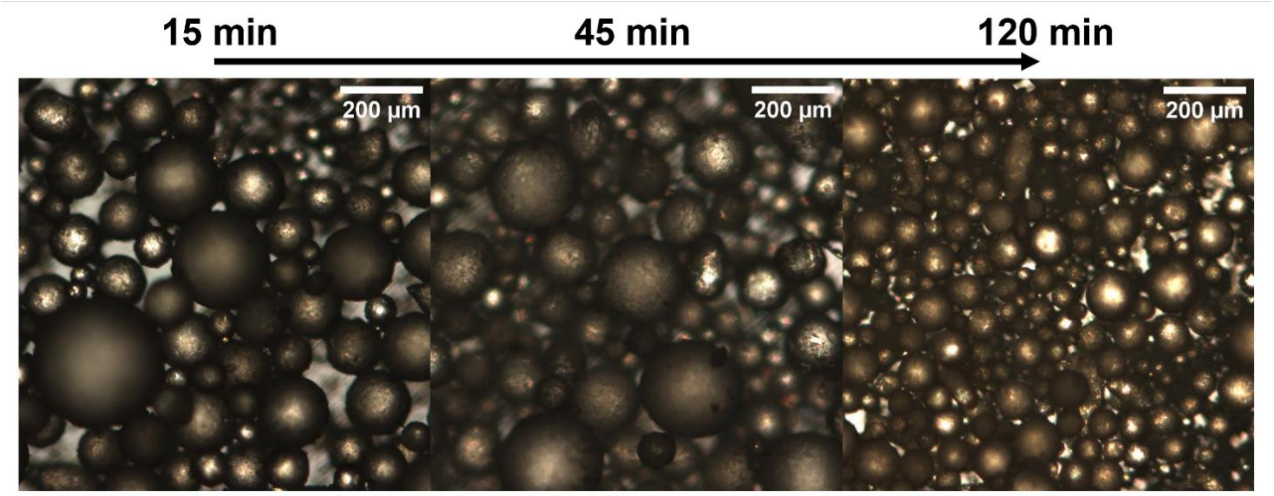

Figure S5 – OM images of the colloidosomes prepared by the emulsification process between SiO_2-nm and paraffin wax at a SiO_2-nm/wax ratio of 0.033 in the presence of CTAB at different times.

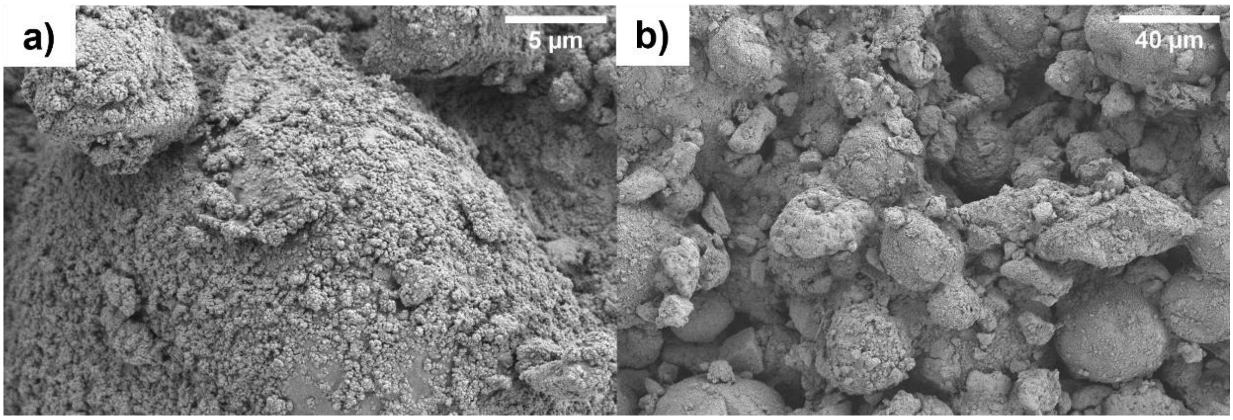

Figure S6 – SEM images at higher (a) and lower (b) magnifications of the colloidosomes prepared by the emulsification process between SiO_2-nm and paraffin wax in the presence of CTAB at a SiO_2-nm/wax ratio of 0.033.

Table S4 – The measured diameters and PDI values of the colloidosomes prepared with SiO_2-nm at SiO_2/wax ratio of 0.033 at different emulsification times. The reported diameters are an average of 50-80 colloidosomes and the PDI is calculated as $\sqrt{(\sigma/d)}$ where σ is the standard deviation and d the average diameter.

**Time (min)**	15	45	120
**Diameter (µm)** *	126 ± 87	126 ± 91	72 ± 29
**PDI**	0.83	0.85	0.64

*The reported diameters are an average of 50 colloidosomes.

S3. Study of SiO_2 colloidosomes in ethanol/water mixture

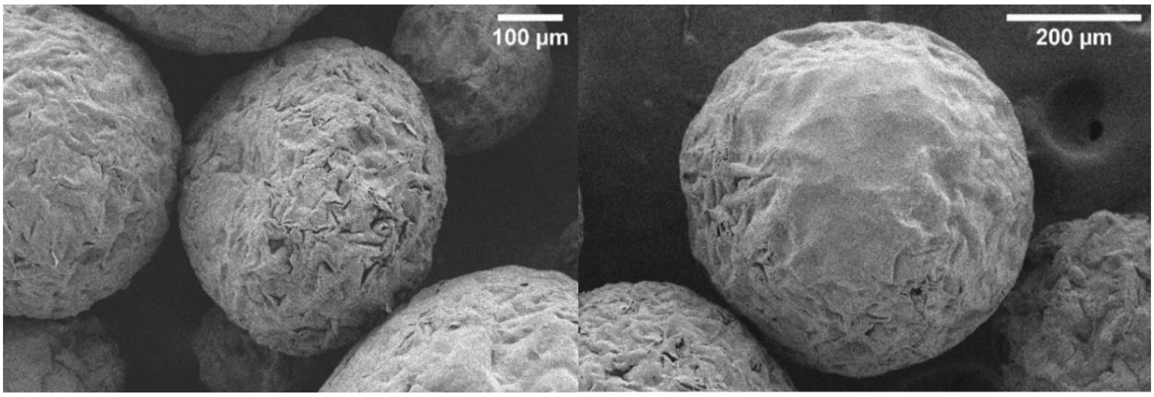

Figure S7 – SEM images at different magnifications of the SiO_2-µm/wax colloidosomes prepared at 0.033 SiO_2/wax ratio after being redispersed in EtOH.

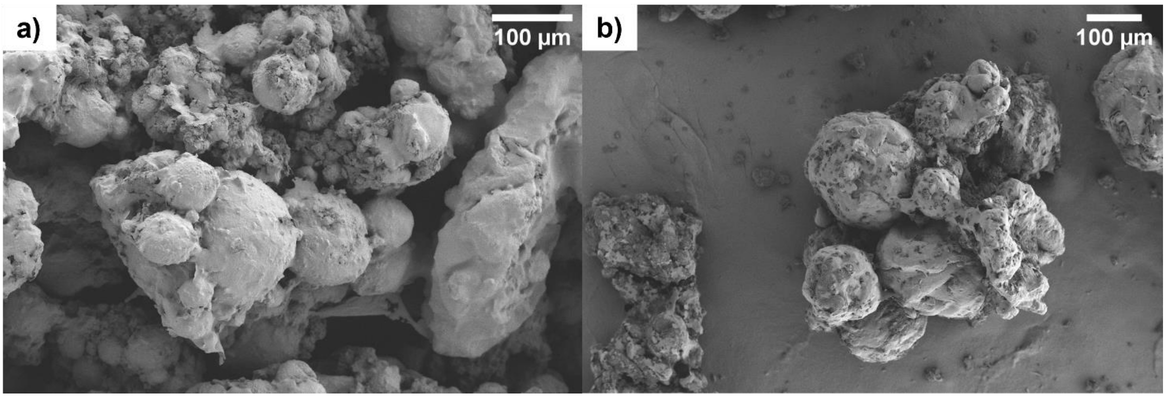

Figure S8 – SEM images of the SiO_2-nm/wax colloidosomes at SiO_2/wax ratio of a) 0.033 and b) 0.159 after being redispersed in EtOH.

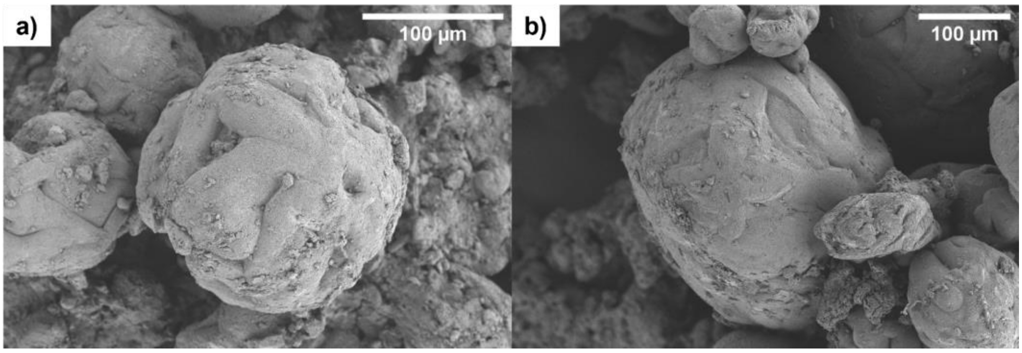

Figure S9 – SEM images of the SiO_2-nm/wax colloidosomes at 0.159 SiO_2/wax ratio being redispersed in H_2O/EtOH a) 70/30 and b) 50/50.

S4 Functionalization of silica particles with APTES

In order to understand the optimal conditions for the selective functionalization of silica particles adsorbed on paraffin wax, the complete functionalization of SiO_2 (Scheme S1) was investigated by changing specific parameters.

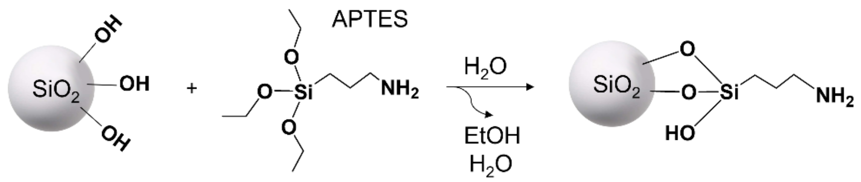

Scheme S1 – The functionalization of SiO_2 with APTES

In detail, the SiO_2-μm functionalization was investigated by changing:

1) EtOH/H_2O solvent ratio (50/50, 30/70)
2) Reaction time (6 h, 24 h, 48 h) and temperature (rt and 35 °C)
3) APTES:OH stoichiometry (1:2, 1:1 and 1.5:1)
4) Catalysis (HCl and NH_3)
5) Ammonia concentration (0.0044 M, 0.0088 M and 0.022 M)
6) Silane (APTMS and APTES)

As a result, the amount of grafted APTES molecules is low independently of the functionalization conditions and varies significantly when repeated. This result could be associated with random variations occurring during the reaction of functionalization.

Small improvements were observed for a stoichiometry APTES:OH 1:1 by employing a 50/50 solvent ratio and an ammonia concentration of 0.0088 M. Therefore, starting from these conditions, the functionalization of SiO_2-nm was investigated (Table S5). For the functionalization of SiO_2-nm, given the mild conditions, the silane was added in a concentration comparable to the one employed for the functionalization of SiO_2-μm and no ammonia was added.

The resultant APTES-functionalized particles were characterized by TGA and CHNS analysis in order to determine the amount of bonded APTES (Figure S10).

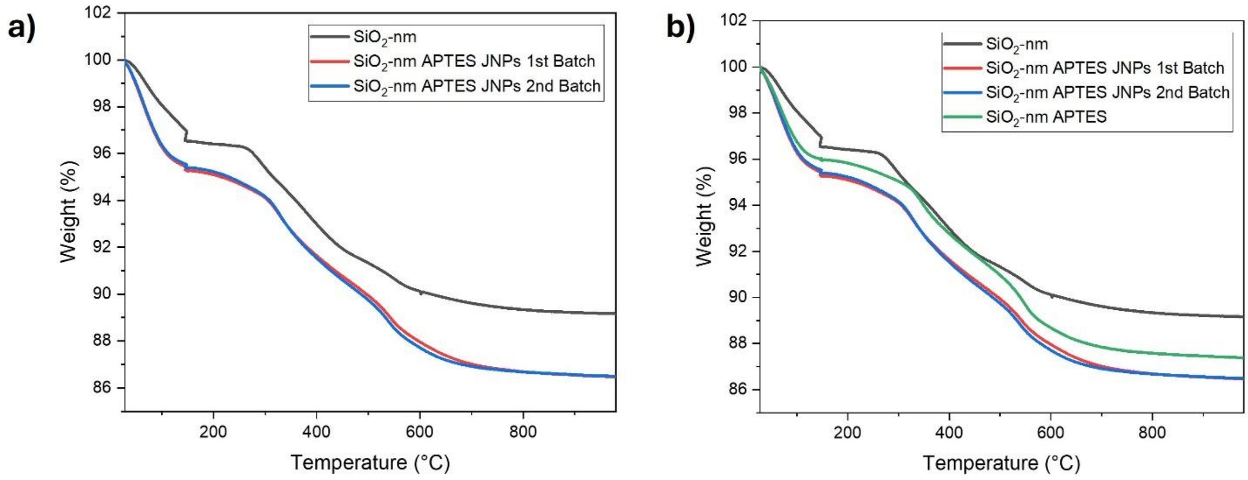

Figure S10. TGA thermograms of the sample SiO_2-nm (synthetic conditions reported in table S5) in comparison with APTES JNPs (a) and APTES NPs (b).

The two reported APTES JNPs batches have been functionalized starting from two different batches of colloidosomes prepared with the same type of silica (SiO_2-nm)

The TGA graph in Figure S10b is particularly representative because it shows that in between the temperature range of 200 and 300 °C, when the wax is degraded (Figure S2), there is no significant difference between the sample functionalized completely (green line, SiO_2-nm APTES) and the JNPs (red and blue lines). Given the fact that their weight loss trends are very similar but shifted (because of the initial water weight loss) we can be sure that all the possible wax present in samples APTES JNPs is completely removed after washing with toluene.

In detail, the APTES wt% was determined by considering the registered weight loss as composed by not only the contribute of i) the functionalized APTES molecules but also ii) the unreacted silanol groups present on the surface of silica and iii) the residual hydroxyl groups bonded to APTES. These last two terms are associated with the assumption that one APTES molecule reacts with two surface OH groups thus leaving one ethoxy group that is most probably hydrolyzed in the reactions conditions. All the mentioned contributions are reported in Eq. S4.

$$\Delta wt\%_{150-1000\ °C}^{SiO_2-APTES} = wt\%_{APTES} + wt\%_{OH+OEt} - 2 \cdot \frac{wt\%_{APTES}}{MW_{APTES}} \cdot \frac{1}{2} \cdot MW_{H_2O} + \frac{wt\%_{APTES}}{MW_{APTES}} \cdot \frac{1}{2} \cdot MW_{H_2O} \quad \text{(Eq. S4)}$$

Where:

- $\Delta wt\%_{150-1000\ °C}^{SiO_2-APTES}$ is the weight loss registered between 150 and 1000 °C for SiO_2-APTES;
- $wt\%_{APTES}$ is the percentage by weight of APTES bonded to SiO_2;
- MW_{APTES} is the molecular weight of bounded APTES (58 g/mol);
- MW_{H_2O} is the molecular weight of H_2O.

By rearranging the equation, the $wt\%_{APTES}$ was obtained (Eq. S5) as reported in Table S6.

$$wt\%\ \text{APTES} = \frac{\Delta wt\%_{150-1000\ °C}^{SiO_2-APTES} - wt\%_{OH+OEt}}{\left(Mw_{APTES} - \frac{1}{2}Mw_{H_2O}\right)} \times Mw_{APTES} \quad \text{(Eq. S5)}$$

Table S5- The reaction parameters investigated for the preparation of APTES-SiO_2-nm

**Parameter**	**SiO_2-nm**
**EtOH/H_2O**	5/5
**Temperature**	rt
**[NH_3]**	0
**Time**	24 h

Table S6 – The parameters obtained by TGA and the resulting APTES wt% for APTES-functionalized SiO_2-nm.

**Sample**	**$\Delta wt\%_{SiO_2}^{TGA}$**	**$\Delta wt\%_{SiO_2-APTES}^{TGA}$**	**APTES wt%**
**SiO_2-nm**	7.15	8.31	1.38

A comparable estimation can be made by starting from the C% or the N% registered by CHNS. In detail, starting from the percentage of carbon and nitrogen contained in a sample of SiO_2 and SiO_2-APTES the APTES wt% can be calculated (Eq. S6 and S7).

$$wt\%\ APTES\ (C)\ = \left(\frac{\%C_{SiO_2-APTES} - \%C_{SiO_2}}{3 \cdot MW_C} \right) \times MW_{APTES} \quad \text{(Eq. S6)}$$

$$wt\%\ APTES\ (N)\ = (\frac{\%N_{SiO_2-APTES} - \%N_{SiO_2}}{MW_N}) \times MW_{APTES} \quad \text{(Eq. S7)}$$

where:

- $\%C_{SiO_2-APTES}$ is the percentage of carbon of SiO_2-APTES;
- $\%C_{SiO_2}$ is the percentage of carbon of SiO_2;
- $\%N_{SiO_2-APTES}$ is the percentage of nitrogen of SiO_2-APTES;
- $\%N_{SiO_2}$ is the percentage of nitrogen of SiO_2.
- MW_C is the molecular weight of carbon;
- MW_N is the molecular weight of nitrogen.

Table S7 - CHNS results for the APTES-functionalized SiO_2-nm

Sample	C%	N%	APTES wt% [C]	APTES wt% [N]
**SiO_2-nm**	4.19	1.33	0.97	2.03

As shown in Tables S6 and 7, in the case of SiO_2-nm, even if the reaction was performed at rt, APTES molecules were successfully grafted on silica NPs surface. In detail, for the sample prepared in the absence of any catalyst, the APTES wt% was 1.38 (SiO_2-nm) while if catalyzed, the resultant sample (SiO_2-nm (B)) reached 1.45 wt% according to TGA. It seems therefore that the basic catalyst, at this concentration, does not significantly influence the reaction. At the same time, CHNS analysis shows for both samples a higher presence of carbon (C) and nitrogen (N) compared to bare silica, associated to the APTES presence. These values are partially coherent with the APTES wt% registered by TGA. Differences in the amount calculated from N% can be associated with impurities.

S5 Functionalization of silica colloidosomes with APTES

Table S8 - The parameters registered by TGA and the resulting APTES wt% for APTES-JNPs.

Sample	$\Delta wt\%^{SiO_2}_{150-1000\,°C}$	$\Delta wt\%^{SiO_2-APTES}_{150-1000\,°C}$	APTES wt%
**APTES-JNPs**	7.15	8.43	1.52

Table S9- The CHNS results for the APTES-functionalized SiO_2-nm

Sample	C%	N%	APTES wt% [C]	APTES wt% [N]
**APTES-JNPs**	4.68	1.39	1.76	2.28

S6 Au-JNPs Heterodimer

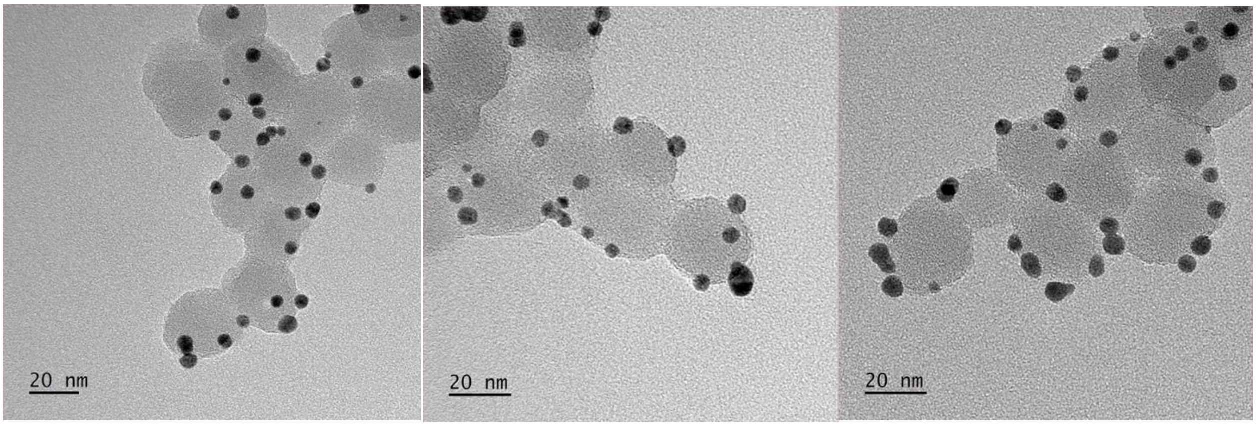

Figure S11 – TEM micrographs of the Janus heterodimer with Au NPs localized only on one hemisphere of APTES-JNPs

It is important to note that the filamentous features observed in the JNPs are similar to those observed in fully APTES-functionalized nanoparticles (Figure S12), indicating that these structures arise from the organic functionalization rather than from wax residues, which are completely removed by the toluene washing step.

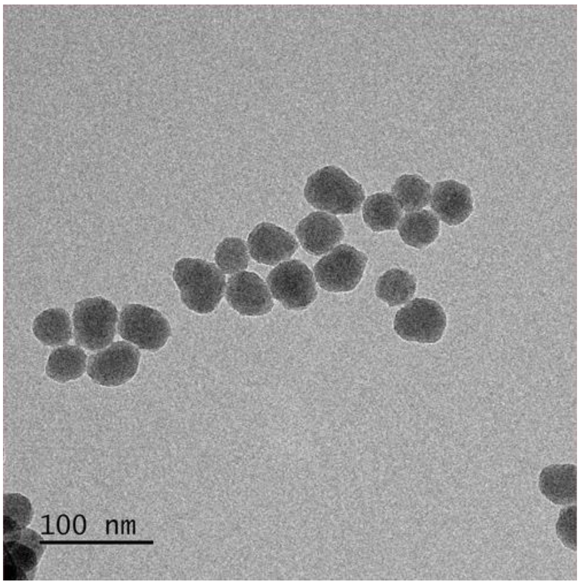

Figure S12 – TEM micrograph of fully APTES-functionalized nanoparticles APTES-NPs (a)

S7 PB and PS grafting of APTES JNPs

The quantification of the grafted polymer PB and PS was performed by combining the information obtained from TGA analysis.

$$\Delta wt\%\,^{Polym-JNPs}_{150-1000\ °C} = wt\%_{Polymer} + wt\%_{APTES} + wt\%_{OH+OEt} - 2\cdot\frac{wt\%_{APTES}}{MW_{APTES}}\cdot\frac{1}{2}\cdot MW_{H_2O} + $$

$$+\frac{wt\%_{APTES}}{MW_{APTES}}\cdot\frac{1}{2}\cdot MW_{H_2O} \quad \text{(Eq. S8)}$$

Eq. S8 can be also expressed in the form of the Eq. S9 in order to directly retrieve the grafted polymer weight percentage.

$$wt\%_{Polymer} = \Delta wt\%\,^{Polym-JNPs}_{150-1000\ °C} - \Delta wt\%\,^{APTES-JNPs}_{150-1000\ °C} \quad \text{(Eq. S9)}$$

Table S10 - The data registered by TGA and the resulting polymer wt% for PS and PB-JNPs.

Sample	$\Delta wt\%\,^{APTES-JNPs}_{150-1000\ °C}$	$\Delta wt\%\,^{Polym-JNPs}_{150-1000\ °C}$	Polymer wt%
**PS-JNPs**	8.43	22.54	14.11
**PB-JNPs**	8.54	20.66	12.12
